# A novel lipid transfer protein from the pea *Pisum sativum*: isolation, recombinant expression, solution structure, antifungal activity, lipid binding, and allergenic properties

**DOI:** 10.1186/s12870-016-0792-6

**Published:** 2016-04-30

**Authors:** Ivan V. Bogdanov, Zakhar O. Shenkarev, Ekaterina I. Finkina, Daria N. Melnikova, Eugene I. Rumynskiy, Alexander S. Arseniev, Tatiana V. Ovchinnikova

**Affiliations:** M.M.Shemyakin and Yu.A.Ovchinnikov Institute of Bioorganic Chemistry, Russian Academy of Sciences, Miklukho-Maklaya str., 16/10, 117997 Moscow, Russia

**Keywords:** Allergen, Antimicrobial activity, Differential gene expression, Garden pea, Heteronuclear NMR spectroscopy, Lipid binding, Lipid transfer protein, *Pisum sativum*, Recombinant expression, Spatial structure

## Abstract

**Background:**

Plant lipid transfer proteins (LTPs) assemble a family of small (7–9 kDa) ubiquitous cationic proteins with an ability to bind and transport lipids as well as participate in various physiological processes including defense against phytopathogens. They also form one of the most clinically relevant classes of plant allergens. Nothing is known to date about correlation between lipid-binding and IgE-binding properties of LTPs. The garden pea *Pisum sativum* is widely consumed crop and important allergenic specie of the legume family. This work is aimed at isolation of a novel LTP from pea seeds and characterization of its structural, functional, and allergenic properties.

**Results:**

Three novel lipid transfer proteins, designated as Ps-LTP1-3, were found in the garden pea *Pisum sativum*, their cDNA sequences were determined, and mRNA expression levels of all the three proteins were measured at different pea organs. Ps-LTP1 was isolated for the first time from the pea seeds, and its complete amino acid sequence was determined. The protein exhibits antifungal activity and is a membrane-active compound that causes a leakage from artificial liposomes. The protein binds various lipids including bioactive jasmonic acid. Spatial structure of the recombinant uniformly ^13^C,^15^N-labelled Ps-LTP1 was solved by heteronuclear NMR spectroscopy. In solution the unliganded protein represents the mixture of two conformers (relative populations ~ 85:15) which are interconnected by exchange process with characteristic time ~ 100 ms. Hydrophobic residues of major conformer form a relatively large internal tunnel-like lipid-binding cavity (van der Waals volume comes up to ~1000 Å^3^). The minor conformer probably corresponds to the protein with the partially collapsed internal cavity.

**Conclusions:**

For the first time conformational heterogeneity in solution was shown for an unliganded plant lipid transfer protein. Heat denaturation profile and simulated gastrointestinal digestion assay showed that Ps-LTP1 displayed a high thermal and digestive proteolytic resistance proper for food allergens. The reported structural and immunological findings seem to describe Ps-LTP1 as potential cross-reactive allergen in LTP-sensitized patients, mostly Pru p 3^+^ ones. Similarly to allergenic LTPs the potential IgE-binding epitope of Ps-LTP1 is located near the proposed entrance into internal cavity and could be involved in lipid-binding.

**Electronic supplementary material:**

The online version of this article (doi:10.1186/s12870-016-0792-6) contains supplementary material, which is available to authorized users.

## Background

Plant non-specific lipid transfer proteins (LTPs) assemble an ubiquitous family of small cationic proteins subdivided into two families with molecular masses of ~9-10 kDa (LTP1) and ~7 kDa (LTP2). A biological role of these proteins in plants is still a matter of discussion, although they are proposed to be involved in angiosperms fertilization, adhesion of pollen, somatic embryogenesis, lipid metabolism, formation of the cuticle, cell death, plant signaling and protection of plants against biotic and abiotic stresses [1–4]. Some lipid transfer proteins, for example OsLTPL36 in rice, were shown to be essential for seed development [5]. A number of LTPs exhibit antimicrobial and antiviral properties, as well as antiproliferative activity against tumor cells *in vitro* [6]. Recently, the lipid transfer protein displaying antinociceptive activity was isolated from noni (*Morinda citrifolia* L.) seeds [7].

LTP genes occur in all land plants from the most primitive liverworts and mosses to tracheophytes, but were not found in lower plants such as algae. In this regard, it is suggested today that LTPs have been upraised in plants after their transition from water to land, i.e. about 450 million years ago [8]. These proteins are encoded by large gene families, and it is believed that the genes of multiple LTP isoforms performing different functions during evolution have been introduced by a number of successive duplications of the ancestral gene with subsequent mutations. Diversification of isoform functions is a powerful tool of plant defense system. For example, it is known, that substitution of a single amino acid residue in the mature protein sequence resulted in substantially different antifungal profile [9].

Many LTP1s manifest oneself as important food pollen and latex allergens responsible for allergic reactions. Structural, functional, and immunological studies of novel plant LTPs deepen our knowledge concerning molecular mechanisms of their antimicrobial action, lipid-binding activity, and allergenicity and open the door to practical application of LTPs in medicine and agriculture. It is worth emphasizing that modern diagnostic kits as well as effective vaccines for allergen-specific immunotherapy can be developed on the basis of natural and recombinant plant LTPs and used as a means to reduce immune reactivity and prevent allergic reactions.

The *Fabaceae Leguminosae,* or *Papilionaceae*, better known as the legume, bean, or pea family, is the third largest family of angiosperms after *Orchidaceae* (orchids) and *Asteraceae* (daisies, sunflowers), and the second one after *Poaceae* (grasses) in terms of agricultural and economic importance. Today legumes are an increasingly invaluable food source which is widely distributed around the world due to its high nutritional benefit and unpretentiousness to the cultivation conditions. However, legumes are often cause different allergic reactions. For example, among Spanish children younger than 5 years old, sensitization to legumes such as pea, bean, lentil, and chickpea has the fifth frequency of food allergy occurrence [10]. Legume allergy is also prevalent in Asian countries, particularly in India, where chickpea is a major food allergen [11]. So far, several LTP1 allergens were isolated from the *Fabaceae*: Ara h 9, Ara h 16, and Ara h 17 from the peanut *Arachis hypogaea* [12], Len c 3 from the lentil *Lens culinaris* [13], and Pha v 3 from the bean *Phaseolus vulgaris* [14]. By now two allergens were isolated from the garden pea and registered in the WHO/IUIS Allergen Nomenclature Sub-committee database (http://www.allergen.org/): Pis s 1 (Vicilin, 47 kDa) and Pis s 2 (Convicilin, 97 kDa). In this paper we report isolation, recombinant expression, solution structure, antifungal activity, lipid binding, and allergenic properties of a novel LTP from the garden pea *Pisum sativum*.

## Results

### Isolation and characterization of a novel pea LTP

A novel lipid transfer protein was isolated from the pea *Pisum sativum* seeds. Due to the high stability of LTPs a heat treatment of the crude extract was used in order to precipitate high molecular mass proteins. Sequential ultrafiltration, cation exchange chromatography, and RP-HPLC were used to purify the protein (Additional file 1A, B). MALDI-TOF-MS analysis of the obtained fractions revealed the presence of a protein with molecular mass of 9400.48 Da (Additional file 2A). N-terminal amino acid microsequencing of the purified protein, designated as Ps-LTP1, revealed the sequence ALSХGTVSADMAPХVTYLQA- having significant homology with that of LTP1 subfamily along with similarity of their CD spectra (Additional file 3). Reduction of Ps-LTP1 in the presence of DTT and subsequent alkylation with iodoacetamide resulted in the molecular mass increase by 464 Da (Additional file 2B). Alkylation of the purified protein without prior reduction did not lead to any molecular mass changes (data not shown). These results demonstrated that Ps-LTP1 contained 8 cysteine residues forming 4 disulfide bonds. A yield of the Ps-LTP1 came up to 1 mg per 100 g of the pea seeds.

### RT-PCR cloning, and sequencing of the pea LTP precursors cDNAs

**Fig. 1:**
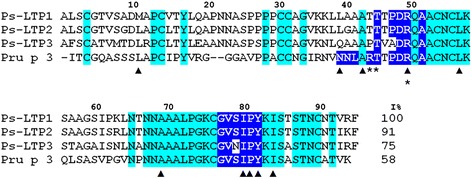
Amino acid sequences of novel pea LTPs and Pru p 3. The conserved residues across all sequences are highlighted in cyan. The residues participating in lipid-binding are marked with a triangles [1, 36]. The residues forming the conformational epitope of Pru p 3 are boxed in dark blue. The residues of Pru p 3 crucial for IgE-binding are asterisked. I% – percentage of sequence identity

By means of RACE strategy two full-length cDNAs (Ps-LTP1-2) and a partial cDNA (Ps-LTP3) encoding the precursors of novel pea LTP1s were determined. Three nucleotide sequences [GenBank accession numbers: KJ569141, KJ569142, and KJ569143 for Ps-LTP1-3, respectively] include 357-360 bp open reading frames encoding the protein precursors of 119-120 amino acid residues (a.a.) long. The sequence analysis was carried out on the SignalP_v4.1 (http://www.cbs.dtu.dk) web server and showed the most probable borders between the signal sequences and the mature proteins. The precursors of three novel pea LTPs include 24–25 a.a. signal peptides and 95 a.a. mature proteins, containing 8 conservative cysteine residues each (Fig. 1).

### Tissue-specific gene expression analysis by quantitative real-time RT-PCR

**Fig. 2:**
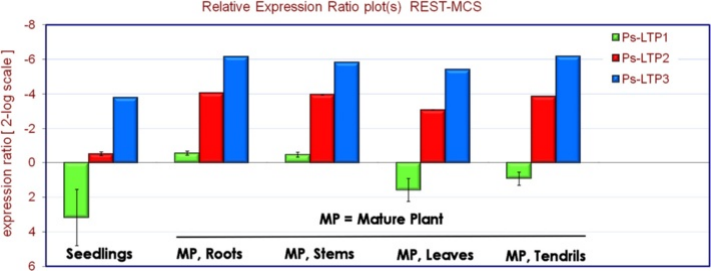
Differential expression profiles of the Ps-LTP1-3 genes in various pea organs. All expression values are normalized to the reference mean of the β-tubulin gene expression. The mRNA levels in different tissue samples were calculated for each gene relatively to its expression in control pea dry seeds before germination

Gene expression profiles of the Ps-LTP1-3 isoforms in different pea organs were studied using real-time RT-PCR. Melting-curve analysis confirmed that the primer pairs for PCR amplification of Ps-LTP1-3 and β-tubulin fragments were specific. Amplification rate for each primer pair was evaluated on the basis of a linear regression slope of a dilution row (Pearson correlation coefficient R^2^ = 0.95–0.99). Relative expression software tool (REST^©^) was used for group-wise comparison and statistical analysis of relative expression results of real-time RT-PCR experiments. REST-MCS was picked out of the several software versions as it allowed to compare up to six conditions of interest with one reference condition for up to ten samples per group. The mRNA levels in different tissue samples were calculated for each gene relatively to its expression in control pea dry seeds before germination (Fig. 2).

Based on the obtained experimental data the Ps-LTP1 gene exhibited high expression level in dry seeds that sharply decreased after germination. Apparently, the protein fulfils its biological role before germination at very early seedling stages. In contrast, expression levels of the Ps-LTP2 and Ps-LTP3 genes increased sharply after germination and remained the same at all tested organs of mature pea plants.

### Molecular characterization of Ps-LTP1

The calculated molecular mass of the mature oxidized Ps-LTP1 (9548.08 Da) differs by 147 Da from the protein *m/z* (9401.48 Da) measured by MALDI-TOF-MS which coincides with the absence of the C-terminal phenylalanine residue. To confirm this suggestion a tryptic digestion of the reduced Ps-LTP1 was performed. The protein trypsinolysis resulted in 14 peptide fragments (Additional file 4A). Molecular masses of the fragments were calculated with PeptideMass tool on the ExPASy online-server (http://web.expasy.org). The calculated values matched well with the measured *m/z* by MALDI-TOF-MS (Additional file 5). The C-terminal fragments containing phenylalanine residue were not found. The structures of the obtained fragments were confirmed by MALDI-LIFT-TOF/TOF-MS microsequencing (Additional file 4B). As a result the complete amino acid sequence of Ps-LTP1 was determined. The primary structure of Ps-LTP1 corresponded to a translated nucleotide sequence of the mature protein without the C-terminal phenylalanine residue [UniProt Knowledgebase accession number: C0HJR7]. Due to the fact that all the purification stages were carried out under ice-cold conditions in the presence of protease inhibitors, it was concluded that an enzymatic cleavage of the C-terminal phenylalanine residue occurred directly in the plant cells. It should be mentioned that two LTP isoforms without C-terminal phenylalanine residues were found earlier in the lentil germinated seeds [15].

### Heterologous expression, purification, and characterization of the recombinant Ps-LTP1 and its ^13^C, ^15^N-labeled analogue

*E. coli* BL21 (DE3) Star™ cells were transformed by pET-His8-TrxL-Ps-LTP1 plasmid containing thioredoxin A (Met37Leu) as a carrier protein. A single Met residue in Ps-LTP1 sequence was replaced with Leu in order to prevent the target protein cleavage by CNBr. It was shown that Met/Leu substitution did not affect the protein structure [16]. The recombinant Ps-LTP1 and its ^13^C,^15^N-labeled analogue were overexpressed in *E. coli*. Decrease of the induction temperature to 26–28 °C resulted in increasing levels of the target fusion proteins in a soluble form. The yields of the recombinant Ps-LTP1 and its ^13^C,^15^N-labeled analogue amounted to 5 and 2.5 mg/L of the bacterial culture, respectively.

CD spectra of the recombinant unlabeled and ^13^C,^15^N-labeled proteins were similar to the CD spectrum of native Ps-LTP1. Antimicrobial activity test and antibody binding assay did not reveal any behavior differences between native (without the C-terminal Phe) and recombinant (full-length) forms of the Ps-LTP1.

### Biological activity of Ps-LTP1

**Fig. 3:**
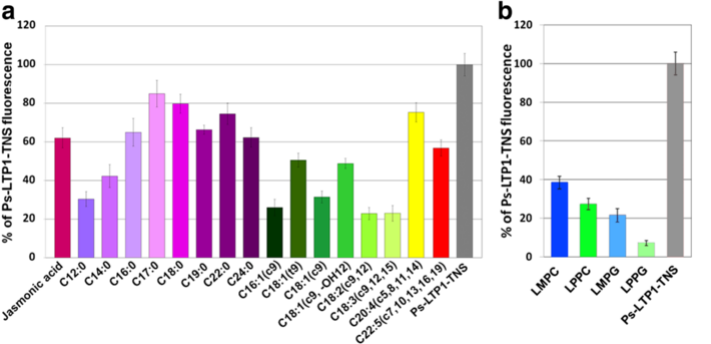
Effect of FAs and JA (**a**) or lysolipids (**b**) on the fluorescence level of the Ps-LTP1-TNS complex. FAs (18 μM) or JA (8 μM) or lysolipids (8 μM) and TNS (3.5 μM) were incubated together for 1 min and then Ps-LTP1 (4 μM) was added. Each experiment was performed in triplicate. The results are expressed as the mean values (±SD) of the percentage of the fluorescence using the Ps-LTP1-TNS complex without lipids as a control

An ability of the recombinant Ps-LTP1 to interact with different lipids was estimated using the TNS fluorescent probe. Saturated and unsaturated fatty acids (FAs) jasmonic acid (JA), and lysolipids were used as test compounds. It is known that a fluorescence of TNS rises sharply upon binding with a hydrophobic cavity of a protein [17]. No significant interaction between the tested lipids and TNS was detected. It was shown that Ps-LTP1 bound all the tested lipids with different efficiencies (Fig. 3a, b), as well as the TNS lipophilic probe. When Ps-LTP1 was added to the mixture of TNS and a lipid, the last one was able to compete with TNS for binding to the protein. It was observed that unsaturated FAs displaced the TNS with the higher efficiency than saturated FAs. Linoleic (C18:2, all-*cis*-9,12) and linolenic (C18:3, all-*cis*-9,12,15) acids competed with TNS most effectively, both leading to 23 % of the initial TNS fluorescence. At the same time, stearic (C18) and margaric (C17) acids displaced the TNS probe with the lowest efficiencies (80 and 85 % of the control fluorescence, respectively). Despite a relatively small size of the molecule, JA displaced TNS with moderate efficiency (62 % of the control fluorescence). Lysolipids competed with TNS with high efficiencies, especially negatively charged LMPG (C14) and LPPG (C16), leading to 22 and 7 % of the control fluorescence, respectively. According to the obtained data Pru p 3 can bind a broad spectrum of saturated and unsaturated FA, JA and lysolipids similarly to Ps-LTP1 (data not shown).

Antimicrobial activity of Ps-LTP1 against gram-negative bacteria *A. tumefaciens* and *P. syringae* gram-positive bacterium *C. michiganensis*, and phytopathogenic fungi *A. alternata*, *A. niger*, A*. versicolor*, *F. oxysporum*, *F. solani,* and *N. crassa* was studied. It was shown that Ps-LTP1 possessed antimicrobial activity (Table 1). *F. solani* and *F. oxysporum* were the most sensitive test microorganisms to Ps-LTP1. It was shown that Ps-LTP1 inhibited spore germination and slowed down hyphae elongation of phytopathogenic fungi, but did not induce its morphological distortions.

**Table 1:** Antimicrobial activity of Ps-LTP1

Test microorganisms	IC_50_, μM
Bacteria	
* Agrobacterium tumefaciens*, strain A281	>40
* Clavibacter michiganensis*, strain VKM Ac-1144	>40
* Pseudomonas syringae*, strain VKM B-1546	>40
Fungi	
* Alternaria alternata,* strain VKM F-3047	na^a^
* Aspergillus niger*, strain VKM F-2259	40
* Aspergillus versicolor,* strain VKM F-1114	na^a^
* Fusarium oxysporum*, strain ТСХА-4	20–40
* Fusarium solani*, strain VKM F-142	10–20
* Neurospora crassa*, strain VKM F-184	40

^a^ *na* not active

An ability of Ps-LTP1 to induce a leakage of large unilamellar vesicles (LUVs) containing encapsulated calcein was estimated. The leakage was shown to depend on the protein concentrations for the vesicles composed of the anionic phospholipid POPG (Additional file 6). On the other hand the leakage was insignificant at all the tested protein concentrations for POPC and POPC/POPG (1:1 molar ratio) liposomes (data not shown).

### Conformational heterogeneity and secondary structure of Ps-LTP1

More than 140 cross-peaks of the backbone ^1^H^15^N groups were identified in the 2D ^15^N-HSQC spectrum of Ps-LTP1 (Additional file 7A) instead of 84 expected (95 residues excluding 10 prolines and one N-terminal residue). The analysis of 3D ^15^N-NOESY-HSQC spectrum revealed the presence of exchange H^N^-H^N^ cross-peaks between some of the spin systems (Additional file 7B). These observations disclose the presence of the two structural forms of the protein in solution caused by the slow (on the NMR time scale) conformational exchange process. (Please note that homogeneity of the Ps-LTP1 preparation was proved by biochemical methods). Analysis of intensities of diagonal and exchange cross-peaks in the 3D NOESY spectrum (τ_m_ 80 ms) permitted to estimate the relative population of the two forms of the protein (~85:15) and the rate of the exchange between them (K_EX_ ~ 10 s^−1^). This rate corresponds to characteristic time of the exchange process ~ 100 ms.

**Fig. 4:**
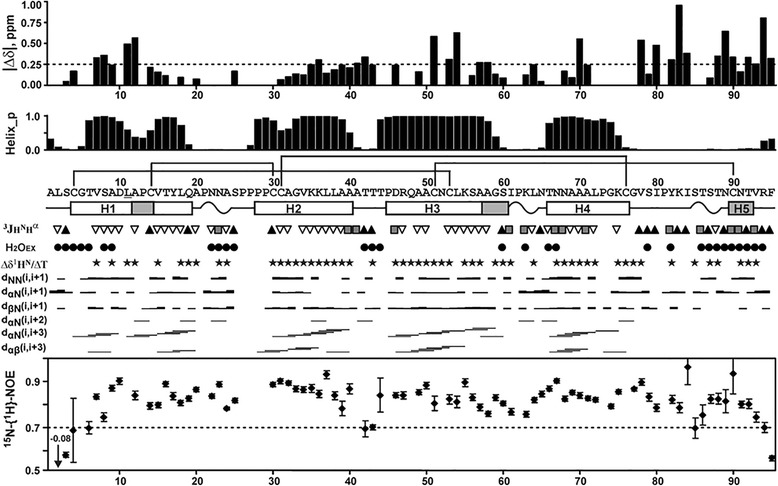
NMR data define secondary structure, dynamics and conformational heterogeneity of Ps-LTP1 in solution. From top to bottom: (δΔ) Root from the sum of squared differences in ^1^H^N^ and ^15^N^H^ chemical shifts for the two structural forms. Differences in the ^15^N chemical shifts were scaled by factor 0.2. The arbitrary taken cutoff value (0.25 ppm) shows residues subjected to large-amplitude motions in ms time scale. (Helix_p) Probability of helix conformation calculated in TALOS+. The secondary structure of Ps-LTP1 is shown below the protein sequence. The α- and probable 3_10_-helical elements are shown by white and gray bars, respectively. The helices of the protein are numbered sequentially (H1-H5). The β-turns are denoted by wavy lines. The site of Met11Leu replacement is underlined. (^3^J_H_^N^_H_^α^) Large (>8 Hz), small (<6 Hz) and medium (others) J-couplings are indicated by the up pointing black-filled triangles, gray-filled squares, and down pointing open triangles, respectively. (H_2_O_EX_) Amide protons which demonstrate fast exchange with water protons are shown by filled circles. The corresponding cross-peaks on the water frequency were observed in the 3D 15N-TOCSY-HSQC spectrum (τ_m_ = 80 ms). (Δδ^1^H^N^/ΔT) Black-filled stars denote amide protons with temperature gradients less than −4.5 ppb/K. NOE connectivities observed in the 80 ms 3D NOESY spectra are denoted as usual. Steady-state ^15^N-{^1^H}-NOE values are shown on the bottom of the figure. Residues displaying NOE < 0.7 are subjected to enhanced motions in ps-ns time scale

The almost complete ^1^H ^13^C, and ^15^N resonance assignment was obtained for the major structural form of Ps-LTP1. The spatial structure and dynamics of this form was studied by heteronuclear NMR spectroscopy. The characteristic d_αN_(i,i + 3) and d_αβ_(i,i + 3) NOE contacts, temperature coefficients of amide protons and values of ^3^J_H_^N^_H_^α^ coupling constants revealed that this form of the protein involved four relatively long helical elements of the secondary structure: Cys4-Gln19 (H1), Pro29-Ala40 (H2), Pro45-Ser60 (H3), and Thr66-Cys76 (H4) (Fig. 4). The H1-H4 elements have predominantly α-helical conformation, but the turns of 3_10_-helix were observed in the middle of H1 (at the level of Pro13) and at the C-terminal end of H3 (Ala58-Ser60, Fig. 4). In addition, the protein molecule involves a long C-terminal tail (Gly77-Phe95) which incorporates β-turn and isolated turn of 3_10_-helix (helical element H5, Cys90-Thr92).

Due to low population in solution only limited ^1^H and ^15^N resonance assignment (58 spin systems) was obtained for the minor structural form of Ps-LTP1. Very frequently the similar slow conformational exchange in protein molecules is caused by *cis-trans* isomerization of the Xxx-Pro peptide bonds. The Ps-LTP1 molecule accommodates 10 Pro residues and four of them form one sequential fragment (Pro26-Pro29). The obtained ^13^C chemical shifts for Pro residues and observed sequential NOE cross-peaks revealed that in the major structural form of the protein the all Xxx–Pro peptide bonds had *trans*-configuration. At the same time, the maximal differences in chemical shifts between the two protein forms (Fig. 4, top) were observed in the middle parts of the helices H1, H3, and H4, and in the *C*-terminal tail. These regions contain only two proline residues (Pro13 and Pro81). Thus the observed exchange process could be originated by *cis-trans* isomerization of the Ala12-Pro13 or Ile80-Pro81 peptide bonds, or by some other structural rearrangement (e.g. by change in the conformation of Cys51-Cys90 disulfide bridge). The available experimental data do not permit to distinguish between these possibilities.

The observed conformational exchange process also could be caused by slow binding/unbinding of some hydrophobic molecule presented in the sample. This possibility seems to be quite unlikely. First of all the protein was purified by several chromatographic steps (including two-step RP-HPLC) which removed any traces of hydrophobic impurities. Second, if this hypothetic ligand was tightly bound to the protein during purification procedure, it should be detected by mass spectrometry, but this is not the case. Moreover, we did not observe any surplus aliphatic signals and NOE contacts in the NMR spectra of Ps-LTP1. The above data testified that NMR sample contained Ps-LTP1 in the unliganded form.

### Spatial structure of the major structural form of Ps-LTP1

**Fig. 5:**
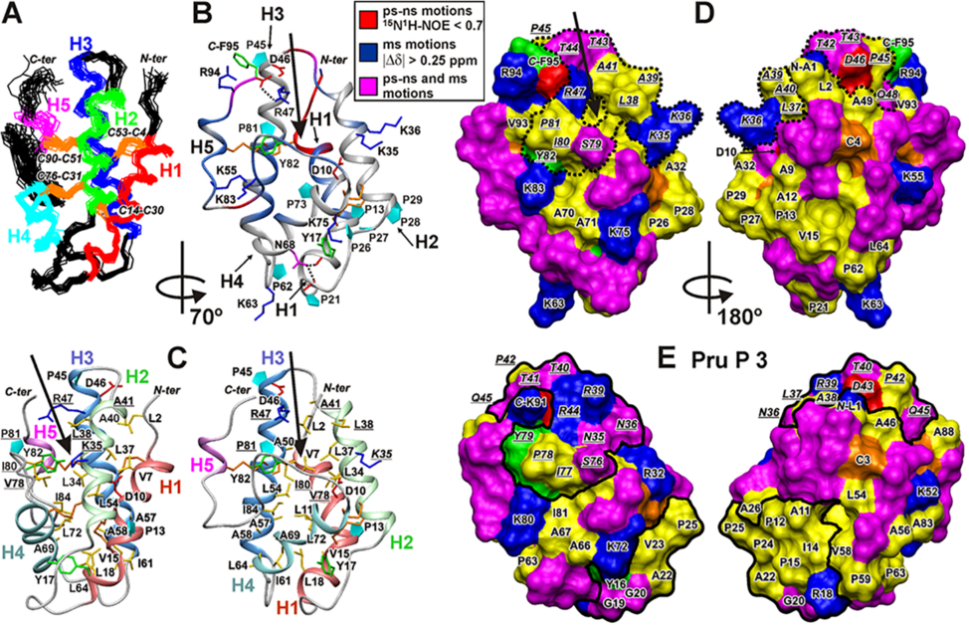
Spatial structure and backbone dynamics of the major structural form of Ps-LTP1 in solution. **a**. The sets of the best 20 structures are superimposed over the backbone atoms in regions with well-defined structure (Cys4-Cys76). The disulfide bonds are shown in orange. The helices H1-H5 are color coded. **b.** Ribbon representation of the Ps-LTP1 spatial structure. The ribbon is colored according to obtained dynamical NMR data (see the legend in Fig. 3). The positively charged (Arg, Lys, N-terminal amide), negatively charged (Asp, C-terminal carboxylic group), and aromatic (Phe/Tyr) side chains/moieties are in blue, red, and green, respectively. The Pro residues are shown by cyan plates. The hydrogen bonds between side chain Asn68 (magenta) and CO groups of Tyr17 and Pro21 (red cylinders), and ionic bridge (Arg47 – *C*-terminus) are shown by broken lines. **c.** Two-sided view of the Ps-LTP1 spatial structure. The side chains of hydrophobic residues (Ala/Ile/Leu/Val) which form the internal cavity are colored in yellow. The residues that form the entrance into the internal hydrophobic cavity are marked by underlined lettering. The helices H1-H5 are color coded. **d**, **e**. Two-sided views of the surfaces of Ps-LTP1 and Pru p 3 (PDB ID 2ALG, [28]) molecules, superimposed over C^α^ atoms of the eight conserved Cys residues. The color code is similar to one used at the other panels, except that Pro residues are colored in yellow. The two IgE epitopes (one conformational Asn35-Ala46/Ser76-Tyr79 and one sequential Ala11-Pro25) are shown on the surface of Pru p 3 by thick black lines. The Ps-LTP1 region homologues to conformational epitope of Pru p 3 is shown by dotted line. The corresponding residues on the both molecules are shown by italic and underlined lettering. Ps-LTP1 molecules shown on the panels **b**, right **c**, and left **d** have identical orientation. The expected entrance into internal hydrophobic cavity is shown by arrow

The set of 20 spatial structures (Fig. 5a) for the major form of Ps-LTP1 was calculated from the available NMR data (Additional file 8). The protein is stabilized by four disulfide bonds (Cys4-Cys53 Cys14-Cys30, Cys31-Cys76, Cys51-Cys90) and 46 backbone-backbone hydrogen bonds associated with elements of secondary structure. Apart from these bonds, the protein structure are stabilized by additional H-bonding and electrostatic interactions (Fig. 5b). For instance, two possible hydrogen bonds (HNδ21 Asn68 – CO Tyr17 and HNδ22 Asn68 – CO Pro21) control the spatial arrangement of helix H4 relative to the loop connecting H1 and H2 helices (H1-H2 loop). The capping interaction (HN Asp46 – Oγ1 Thr43) stabilizes the N-terminus of H3 helix. In addition, the possible ionic bridge between guanidinium group of Arg47 and the C-terminal carboxylic group control the relative position of the C-terminal tail relative to H2-H3 loop.

The spatial structure of the protein is fairly good defined in the region of the conserved helical core (H1-H4) that stabilized by disulfide bonds (RMSD ~ 0.8 Å over backbone atoms of the Cys4-Cys76 residues, Additional file 8). The reduced convergence of the calculated structural ensemble in the N-terminal region (Ala1-Cys4) and C-terminal tail (Gly77-Phe95) (Fig. 5a; Additional file 8) is probably connected with the enhanced intramolecular mobility of these segments (Fig. 5b). Indeed the residues from the C-terminal tail demonstrate largest influence of the observed ms time scale conformational fluctuations (Fig. 4 top, see above). In addition, the measured steady-state ^15^N-{^1^H} heteronuclear NOE values (Fig. 4 bottom) revealed enhanced ps-ns time scale mobility at the N- and C-termini of the molecule.

Similarly to other LTP1s in the determined Ps-LTP1 spatial structure the helix H1 demonstrates a pronounced kink (58 ± 5°) at Pro13 (Fig. 5c, Additional file 9). As a result, the parallel bundle formed by helices H1-H3 adopts an overall boat-like shape. The interior of this cavity is lined with apolar side chains and shielded from the aqueous environment by the H4 helix and the C-terminal tail. This hydrophobic pocket, having relatively large volume (1000 ± 300 Å^3^), may play a role in binding of lipids and other hydrophobic molecules. The entrance into the internal hydrophobic cavity (marked by arrow at Fig. 5) is formed by two conservative fragments, located at the H2-H3 loop (Leu38-Arg47) and at the C-terminal tail (Ser79-Tyr82).

In contrast to the inner surface the outer surface of Ps-LTP1 is lined mostly by polar and charged residues (Fig. 5d). The several small apolar patches are formed by side chains of not too hydrophobic residues (mostly Ala and Pro). The largest hydrophobic patch involves residues from H1 and H2 helices, and H1-H2 loop (Ala12-Ala32 fragment, Fig. 5d). Interestingly, the charged residues are distributed non-uniformly on the outer surface of Ps-LTP1. The one side of the molecule (Fig. 5d, left), which involve entrance to the hydrophobic cavity, accommodates all charged residues, except Lys55 and Lys63 protruding to the other side. Only one charged side chain of Ps-LTP1 has virtually no contact with the outer surface. The positively charged residue (Asp10) is burred on the interface between H1 and H2 helices (Fig. 5d, right). Interestingly, the homologues Lc-LTP2 protein from *Lens culinaris* has similar spatial position of Asp10 (Additional file 9).

### Thermal stability of the Ps-LTP1 spatial structure

One of the steps used during purification of the native Ps-LTP1 involved prolonged heating at 80 °C. To investigate the changes in the spatial structure of the protein upon heating the temperature dependence of the secondary chemical shifts of amide protons (Δδ^1^H^N^) was analyzed (Additional file 10). The 20-70 °C temperature range was used; higher temperatures were not available due to instrumental limitations. It is well known that Δδ^1^H^N^ values depend from secondary structure of the protein and could be used as indicators of relative strength of hydrogen bonding at the amide groups [18].

It was found that upon increase in temperature the resonances of the residues, which are strongly influenced by the ms time scale fluctuations, became unobservable. Probably, the increase in the rate of the exchange process with temperature change it regime from slow to intermediate on the NMR time scale. On the other hand, the Δδ^1^H^N^ values for the residues, which are weakly affected by the conformational exchange, have linear temperature dependences (Additional file 10B). The majority of amide protons from the H1-H4 helices demonstrated quite low temperature gradients (< −4.5 ppb/K, Fig. 4) and even at the higher temperature have negative Δδ^1^H^N^ values, which is indicative of the helical conformation (Additional file 10A). Thus one could conclude that neither secondary structure, nor the overall spatial arrangement of Ps-LTP1 molecule are changed upon increase of the temperature.

### In silico analysis for the prediction of allergenicity and cross-reactivity

World Health Organization (WHO), the Food and Agriculture Organization (FAO) with the Codex Alimentarius Commission proposed guidelines for assessment of the potential allergenicity of proteins [19]. According to these guidelines a protein is potentially allergenic if it shares with known allergen either a match of 6 consecutive amino acid residues or an identity of >35 % over a 80 amino acids window [20]. This algorithm is used primarily for identifying allergic cross-reactivity when a high degree of allergens similarity (more than 50 %) is observed [21].

A sequence analysis by FARRP showed that Ps-LTP1 has more than 47 % identity to more than 31 allergenic LTPs across 80 a.a. window. It shares 81 % identity with the lentil allergen Len c 3 69 %—with the peanut Ara h 9, 68 %—with the green bean Pha v 3, and 60 %—with the peach Pru p 3 over a 80 a.a. window (Additional file 11). It was found, that Ps-LTP1 also shares at least one identical 6-amino-acid stretch with 31 allergenic LTPs. Most of the matches were observed for Len c 3, Pha v 3, Ara h 9, and Pru p 3 (32, 20, 17, and 16 matches, respectively). Presumable allergenic properties were also confirmed by using other web tools, such as AllergenFP, AlgPred and SDAP.

Three sequential IgE epitopes were previously characterized in Pru p 3 (see Fig. 5e) [22]. Two of them form conformational epitope (Asn35-Ala46/Ser76-Tyr79) which includes three key amino acid residues (Arg39 Thr40 and Arg44) providing IgE-binding capacity. Two of the above mentioned key residues—Thr43 and Arg47 (corresponding to Thr40 and Arg44, respectively, in case of Pru p 3) are present on the Ps-LTP1 surface, while positively charged Arg39 is replaced by polar Thr42 (Fig. 5d, e). Thus, there are some differences in the charge distribution over the region of the presumable conformational IgE epitope Thr42-Ala49/Gly77-Tyr82, but the region has in whole the similar physical properties (protuberant position, clustering of the charged moieties, etc.) with main epitope of Pru p 3 (Fig. 5d, e).

### Simulated gastrointestinal proteolysis

**Fig. 6:**
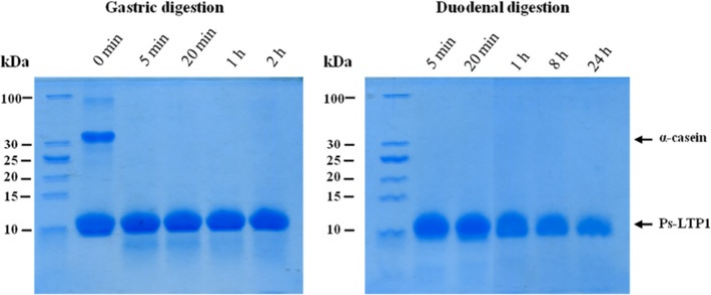
SDS-PAGE analysis of *in vitro* cleavage mimicking gastric and duodenal digestions of Ps-LTP1

The Ps-LTP1 in vitro fragmentation by enzyme cleavage mimicking gastrointestinal digestion was analyzed by SDS-PAGE under reducing conditions (Fig. 6). As an internal control α-casein from bovine milk (Sigma) was added to monitor an enzyme activity. It was shown that Ps-LTP1 displays a remarkable resistance to gastric digestion with pepsin for 2 h (a cleavage yield was 19 %). At the same time, α-casein completely degraded by pepsin within the first 5 min. Upon duodenal digestion, moderate degradation of Ps-LTP1 (a cleavage yield was 47 %) was observed after 24 h treatment of the protein with the mixture of trypsin and chymotrypsin. Thus, it was shown that Ps-LTP1 is characterized by high stability to gastrointestinal digestion which is typical of food allergens.

### Immunological properties of Ps-LTP1

As previously mentioned the most significant similarity was observed between pea Ps-LTP1 and the lentil allergen Len c 3. To verify the immunological similarity of this proteins, a previously obtained rabbit anti-Len c 3 antiserum [23] was used in western blot and ELISA assays. By using SDS-PAGE under reducing conditions with following western blot, it was shown that polyclonal rabbit anti-Len c 3 IgG interact with the recombinant Len c 3, Pru p 3, and Ps-LTP1 as well as with the native LTP from the pea extract (Additional file 12). By ELISA assays the most significant binding of anti-Len c 3 IgG with Len c 3 was shown (Additional file 13A). The weakest binding was found in case of Pru p 3 which has lower structural similarity with Len c 3 than Ps-LTP1. These results were also confirmed by inhibition assays in which binding of anti-Len c 3 IgG with Len c 3 was suppressed by Ps-LTP1 or Pru p 3 (Additional file 13B). The obtained data showed at least partial similarity of IgG epitopes of all the three LTPs.

**Fig. 7:**
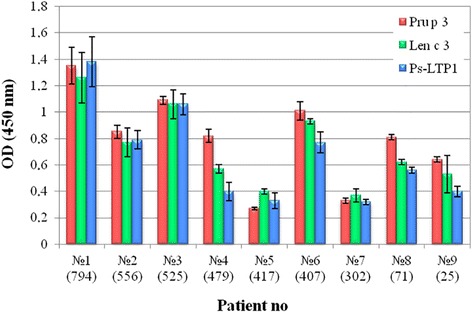
Characterization of sera from patients with food allergy. Total IgE levels are specified in brackets. Data were obtained using 1:2 serum dilutions

The IgE-binding was analyzed in vitro by ELISA assays using the sera from patients with food allergy containing specific IgE to Pru p 3 (Fig. 7). Ps-LTP1 bounds to specific IgE from all the tested sera of Pru p 3 sensitized patients and its immunoreactivity was quite significant, albeit weaker than that exhibited by Pru p 3 and Len c 3. This attests to the fact that Ps-LTP1 might be a novel cross-reactive food allergen.

## Discussion

Plant LTPs comprehend a multigenic family with different genes being expressed at various stages of plant ontogeny. Biosynthesis of multiple LTP isoforms is tissue-specific. Expression of the genes of different LTP isoforms is primarily determined by the environment which makes biosynthesis of multiple LTP isoforms an element of a plant defensive strategy under different abiotic and biotic stresses. The presence of several LTP isoforms was previously demonstrated for the lentil *Lens culinaris* in which a subfamily of 8 novel lipid transfer proteins Lc-LTP1-8 was found [15]. In the present study we confirmed that multiple LTP isoforms are also present in the garden pea *Pisum sativum*. Three new LTP1 isoforms, named as Ps-LTP1-3, were found in the pea seeds, and their cDNAs were determined. All the precursor proteins contained 24–25 a.a. residues signal peptides and 95 residues mature proteins, containing 8 conservative cysteine residues typical of plant LTPs. All the three pea LTPs are similar to each other (91 and 75 % homology of Ps-LTP1 with Ps-LTP2 and Ps-LTP3, respectively) (Fig. 1). Preliminary conclusions can be made from experimental expression profiling of the genes of different Ps-LTP isoforms. (1) Ps-LTP1, an abundant isoform in ungerminated pea seeds, can participate in lipid mobilization during seeds development and germination. It is Ps-LTP1 which was isolated from the ungerminated pea seeds and characterized at the protein level. Its complete amino acid sequence was determined. The protein contains 8 cysteine residues located in highly conserved positions of plant LTP sequences. (2) Expression levels of the Ps-LTP2 and Ps-LTP3 genes rose sharply just only after germination and remained at the same level in different pea organs of mature plant. The latter could argue for more general biological roles of these isoforms in plant physiology, such as participating in cell wall growth, signal transduction or defense against pathogens. Our results afford further molecular insight into possible biological roles of different Ps-LTP isoforms.

Previously two mRNA encoding LTPs have been discovered in the pea seedlings. The first pea LTP [UniProtKB: Q9M7D7] was found only at transcript level [24] and contained 10 cysteine residues which is atypical for plant LTPs. It was shown that the level of its mRNA increased under abiotic stress conditions which confirmed the differential expression of LTP isoforms and their multifunctional properties. The second pea LTP [UniProtKB: O24309] was found also at transcript level [25] and recently detected at a protein level [26]. It was identified by extraction from the pea flour ultra-filtration, reversed-phase fast performance liquid chromatography (RP-FPLC), SDS-PAGE, in-gel digestion by trypsin, and MS/MS tandem mass spectrometry. The protein also has atypical structure and contains 7 cysteine residues. It should be noted that the protein was not isolated from the pea flour, but just identified by tandem mass spectrometry of the tryptic digest. Apparently, its expression in the pea seeds occurs at very low level. That is why we did not isolate this protein, as our purpose was to identify and characterize the main food allergen being constitutively expressed in the pea seeds at high level.

The presently obtained NMR data revealed structural similarities and dissimilarities of the Ps-LTP1 with other plant LTP1s. Analogously to other LTP1s, Ps-LTP1 encompasses four α-helices (H1-H4) which surround the internal hydrophobic cavity probably containing the lipid-binding site, and followed by a long C-terminal tail (Fig. 5). Ps-LTP1 can be fairly good superimposed with the unliganded highly homologous allergen protein Len c 3 from *Lens culinaris* (77 % of identity, PDB ID 2MAL, [27]) as well as with the liganded and less homologous allergen Pru p 3 from peach (58 % of identity, PDB IDs 2ALG and 2B5S, [28]) (Additional file 9). The minimal RMSD between the set of structures, calculated over C^α^ atoms of the eight conserved cysteines, is about 0.6 and 0.8 Å, respectively. At the same time, the comparison revealed significant differences in relative orientation of the interhelical loops and the C-terminal tail (Additional file 9). These differences led to the slightly different packing of the Ps-LTP1 helices and to significant increase of the internal hydrophobic cavity volume. In contrast to other unliganded LTP1s having internal cavities with van der Waals volume from 80 to 800 Å^3^ (the largest cavities were observed in case of Len c 3 [27] and dill Ag-LTP [29]), the lipid-free Ps-LTP1 holds much larger hydrophobic pocket (1000 ± 300 Å^3^). This space is sufficient to accommodate double-chain lipid (~1100 Å^3^) and is similar to the volumes of internal pockets in the liganded LTP1s (from 650 to 1350 Å^3^). The cavities in the partially and fully liganded Pru p 3 have the volumes ~ 500 and 1200 Å^3^, respectively.

The major difference of the Ps-LTP1 from other plant LTP1s is the conformational heterogeneity. In solution the unliganded protein represents the mixture of two conformers with the relative population ~ 85:15 and with characteristic time of interconversion between them on the order of a hundred microseconds. Presently, we are unable to describe the origin of the exchange process that leads to the conformational heterogeneity of Ps-LTP1. It could be connected with the *cis-trans* isomerization of the Ala12-Pro13 or Ile80-Pro81 peptide bonds or with changes in the conformation of the Cys51-Cys90 disulfide bridge, which connect the C-terminal tail to the helical core of the molecule. However, the involvement of residues from all helices (H1-H4) and the C-terminal tail in this process (Figs. 4 and 5b) implies that the corresponding conformational fluctuations are accompanied by the reshaping of the internal hydrophobic cavity. Thus, the minor conformation of Ps-LTP1 probably corresponds to the structure with collapsed internal cavity, having much less volume, similar to that of other unliganded LTP1s structures. To the best of our knowledge, the conformational heterogeneity in the unliganded plant LTPs were not described before.

It is known that plant LTPs belong to pathogenesis-related proteins (PRP) which are involved in systemic acquired resistance protecting plants from pathogens and infections. The defensive role of many LTPs is associated with an ability to inhibit the growth of phytopathogens. However there are some members of LTPs family which do not exhibit any antimicrobial activity or inhibit phytopathogenic growth at relatively high concentrations. A novel LTP isolated from the pea seeds has moderate antifungal activity and is characterized by specificity of its action. The most sensitive to Ps-LTP1 test cultures were fungi of the *Fusarium* genus, causing plant fusariosis, root rot and seedling bligh. It is known that some pea cultivars have high resistance to diseases caused by fungi of the *Fusarium* genus [30]. High resistance of pea to fusariosis might be also a result of high expression level of Ps-LTP1. A putative target of LTP’s antimicrobial action is cytoplasmic membrane [31]. We showed that Ps-LTP1 disturbs the permeability of artificial liposomes, and it is important to note that liposomes composition is of much importance for this action. Ps-LTP1 is able to induce the leakage of POPG liposomes, and this effect is dependent on the protein concentrations, while POPC and POPG/POPC liposomes remain intact. Interestingly, effectiveness of calcein leakage depends on the protein concentrations which correlates to the concentrations used in the antimicrobial assay. Probably, the cationic plant LTPs interact with anionic components of plasma membranes, leading to destabilization and increase of their permeability. The obtained data prove the Ps-LTP1 ability to permeabilize model membranes.

It is known that lipids and their derivatives are involved in various plant physiological processes including membrane biogenesis, cell differentiation, inter- and intracellular signaling. It is supposed that different biological functions of LTPs are based on their ability to bind and transport various lipid molecules. Studies of the lipid-binding properties of LTPs can provide helpful data to understand their biological functions. It is suggested that LTP1s are involved in a transport of hydrophobic monomers which compose the waxy and polymeric cutin layers. Despite a high sequence homology and similarity of their spatial structures, LTPs can exhibit structural differences leading to different affinities to lipids [32]. For example, it was shown that the lentil Len c 3 (the hydrophobic cavity volume is of ~600 Å^3^) has lipid-binding specificity towards unsaturated FAs, while the dill Ag-LTP (~800 Å^3^) has no significant difference in binding of saturated and unsaturated FAs, which could be connected with much larger hydrophobic pocket in Ag-LTP [29]. The determined here spatial structure of Ps-LTP1 holds even larger hydrophobic cavity (~1000 Å^3^) but also has lipid-binding specificity towards unsaturated FAs, which could be linked with the observed conformational heterogeneity of Ps-LTP1. Probably, the specificity to different lipids is “encoded” in the conformation of the minor structural form of Ps-LTP1 that responsible only for ~15 % of the protein presented in solution. In this case the process of lipid-binding to the internal cavity should has compatible timescale or be even slower than the observed exchange process (timescale ~ 100 ms).

LTPs constitute a family of true allergens which can cause a sensibilization to pollen and especially to foods. The isolated from ungerminated pea seeds Ps-LTP1 is a constitutively expressing abundant isoform which may exhibit properties of a true allergen. Food allergens are often characterized by thermal stability and protease resistance. For example, stability to digestion by the gastrointestinal tract, especially by gastric pepsin, has been claimed as a characteristic of a true food allergens [33]. Moreover, their stability to heat treatment also implies the presence of active allergen forms in processed foodstuffs. Due to its extreme resistance to gastrointestinal enzymes, Ps-LTP1 can be evaluated as a potentially severe food allergen. NMR thermal stability experiments and heat treatment of Ps-LTP1 allow to label the protein as heat-resistant one. Moreover, LTPs belong to the most clinically relevant classes of cross-reactive plant allergens. Because of their extremely conserved structures, the LTP1s were found to be responsible for IgE cross-reactions not only between foods, but also between unrelated pollen and plant food allergen sources, and were therefore classified as panallergens. For example, the peach Pru p 3 is a major cross-reactive allergen of LTP1 family which plays a predominant role in sensitization of the majority of allergic patients. We showed that isolated pea LTP has high amino acids similarity to several food allergens, including the lentil Len c 3, peanut Ara h 9, and peach Pru p 3. Commonly, highly conserved molecules having sequence similarities often share surface topology relevant for allergenicity. The known structures of antibody-allergen complexes indicate at least a partial overlapping of the IgG epitopes with IgE ones [34]. We showed that Ps-LTP1 seems to share IgG epitopes with other two IUIS food allergens, namely Len c 3 and Pru p 3, and has similar capacity to bind IgE from patients with food allergies. Similar IgE-immunoreactivity could be caused by existence of similar IgE-binding epitopes on proteins’ surfaces. It is suggested that the IgE-binding region should exhibit both a protuberant local surface and an electrostatically active local molecular domain [22]. In this regard, physical properties of Thr42-Ala49/Gly77-Tyr82 region of Ps-LTP1, which is homologous to conformational epitope of Pru p 3, allow to consider it as most probable IgE epitope of the protein. It should be noted, that the same region was identified also as the major conformational epitope of the wheat Tri a 14 [35]. Interestingly, the residues from this region restrict the entrance into internal hydrophobic cavity in case of both Ps-LTP1 and Pru p 3 and participate in lipid-binding (Fig. 1; Fig. 5d, e). Moreover in case of Pru p 3, above mentioned crucial residue for IgE-binding Arg44 as well as incorporated in IgE-binding region polar Tyr79 are highly conserved for plant LTP1s and presumably responsible also for lipid uptake [1, 36]. This permits to speculate that one region on the surface of plant LTP1s is responsible for both interaction with lipid molecules and IgE-binding.

## Conclusions

In summary we discovered and isolated a novel lipid transfer protein from the garden pea *Pisum sativum*. Here, we developed a procedure for LTP isolation from the pea seeds. Molecular cloning and sequencing of cDNAs encoding the Ps-LTP1 precursor and two other isoforms were performed. Complete amino acid sequence and solution structure of Ps-LTP1 were determined. The bacterial expression system for production of the recombinant Ps-LTP1 and its ^13^C,^15^N-labeled analogue was developed. A biological activity of Ps-LTP1 as well as its lipid-binding capacity were studied. For the first time, the conformational heterogeneity in the unliganded plant LTPs, probably connected with reshaping of the internal lipid-binding cavity, was observed. The reported structural and immunological findings seem to describe Ps-LTP1 as potential cross-reactive food allergen in LTP-sensitized patients, mostly Pru p 3^+^ ones.

## Methods

### Isolation of pea LTP

Seeds (m = 100 g) of the garden pea *Pisum sativum* (the cultivar “Sacharniy 2”, GOST P 52171-2003, series 8374 by “Udachnye semena” company) were powdered, mixed with 300 ml of the extraction buffer (150 mM СН_3_COONH_4_, 200 mM NaCl, 2 mM EDTA, 1.5 % PVPP, pH 5.5, protease inhibitor cocktail (Sigma)), and stirred at 4 °C for 2 h. The extract was clarified by centrifugation (38,000 *g* for 40 min at 4 °C) and dialyzed at 4 °C against 100-fold volume of 50 mM CH_3_COONa, pH 5.3, containing 0.2 mM PMSF. Then a heat treatment at 80 °C for 20 min was performed, and denatured proteins were removed by centrifugation. Supernatant was subjected to a sequential ultrafiltration through YM100 and PM30 membranes using an Amicon pressure cell. The filtrate was loaded onto 1 mL cation exchange column HiTrap SP FF (GE Healthcare). Gradient elution with an increasing concentration of sodium chloride (from 0 to 0.5 M) in 50 mM CH_3_COONa, pH 5.3, at a flow rate of 0.7 ml/min was used. Further purification of pea LTP, designated as Ps-LTP1, was performed by reversed-phase high-performance liquid chromatography (RP-HPLC) on a Vydac C_4_ column (5 μm, 250 × 4.6 mm, Grace) at a flow rate of 0.5 ml/min using a gradient of acetonitrile concentration from 5 to 80 % for 60 min in 0.1 % TFA. Final purification of the protein was performed on a Luna C_18_ column (5 μm, 250 × 4.6 mm, Phenomenex) using the same conditions.

### Amplification, cloning and sequencing of cDNAs encoding the pea Ps-LTPs

Total RNA was isolated from the pea germinated seeds using SV Total RNA Isolation System kit (Promega). RT-PCR was performed with SMART™ RACE cDNA amplification kit (BD Biosciences, Clontech) and Mint RACE cDNA amplification kit (Evrogen).

The degenerate primers for the 3′-RACE were designed corresponding to the conservative regions of nucleotide sequences of LTPs from unrelated plant sources. The first round of 3′-RACE was conducted with the primer No. 1 (TG(T,C)(G,A)G(T,C)GT(C,T)AACAT(T,C)CCTTAC) and the universal adapter primer using the step-down PCR protocol: 1)95 °C - 1 min; 2)25 cycles of 94 °C - 30 s, 50…42 °C - 40 s (the temperature was decreased stepwise by 2 °C every five cycles), 68 °C - 2 min; 3)10 cycles of 94 °C - 30 s, 40 °C - 40 s, 68 °C - 2 min; 4)the final extension for 10 min at 68 °C. The diluted products were amplified by the semi-nested PCR with the primer No. 2 (AACAT(T,C)CCTTAC(A,C)(A,C)GATCAG) and the same adapter primer for 35 cycles at annealing temperature 50°С.

Then 5′-RACE was performed with gene-specific primers complementary to conservative parts of the 3′-untranslated region. The first round with the primer No. 3 (ACAAGAAGATAGGACCACAT) consisted of 25 cycles with annealing at 63–51 °C (the temperature was decreased stepwise by 3 °C every five cycles) and 10 cycles with annealing at 48 °C. The diluted products were reamplified with the primer No. 4 (CCCACTCTCATATACTAGTGA) for 35 cycles at annealing temperature 48 °С.

Finally, another 3′-RACE semi-nested PCR was performed with gene-specific primers complementary to conservative regions of cDNA encoding signal peptides of pea Ps-LTPs. Step-down PCR with primer No. 5 (СССATGAAATTAGCATGTG) was performed using amplification program consisted of 25 cycles with annealing at 65-49 °C (stepwise decrease by 4 °C every five cycles) and 10 cycles with annealing at 45 °C. Then reamplification with the nested primer No. 6 (ATGGTAGTTATTGCGCCT) was performed for 35 cycles at annealing temperature 50°С.

The PCR products were separated in 1.5 % agarose gel, eluted using Cleanup Standard kit (Evrogen) and ligated with pAL2 vector (Evrogen). Then the constructs were used to transform DH-10B *E. coli* (Life Technologies). Nucleotide sequencing analysis was performed using ABI PRISM 3100-Avant (Applied Biosystems).

### Transcripts analysis by quantitative real-time RT-PCR

Garden pea seeds (the cultivar “Sacharniy 2”, GOST P 52171-2003, series 8374 by “Udachnye semena” company) were germinated for 3 days in an incubator on paper soaked with distilled water. After germination seedlings were transferred to glass pots and grown till 35-day-old plants.

For total RNA isolation 3-day-old pea seedlings and tissues from 35-day-old plants were collected separately. Total RNAs from root, leaves, and tendrils were isolated using SV Total RNA Isolation System kit (Promega) according to the manufacturer’s instruction. Total RNA from polysaccharide-rich dry seeds was isolated using MLT method [37]. Total RNA from stems was isolated according to KLC method [38] with some modifications. Briefly, 7.5 ml of extraction buffer (0.25 M NaCl, 0.05 M Tris-HCl (pH 7.5), 20 mM EDTA, 1 % (w/v) SDS, 4 % (w/v) PVPP, 5 % β-mercaptoethanol) was mixed with 7.5 ml of phenol:chloroform:isoamyl alcohol (25:24:1 v/v) in 50-ml Falcon tube. 1 g of frozen pea stems was powdered well by a mortar and a pestle in liquid nitrogen, transferred to the tube and vortexed vigorously. The sample was centrifuged at 12,900 *g* for 2 min at 4 °C, an upper water phase was transferred to a new 50-ml Falcon tube. Phenol traces were removed by extraction with an equal volume of chloroform:isoamyl alcohol (24:1 v/v). The water phase was transferred to a new 50-ml Falcon tube, one tenth volume of 3 M sodium acetate (pH 5.2) and 2.5 volume of cold absolute ethanol were added, than mixed well, and incubated at −20 °C for 30 min. The nucleic acids were precipitated by centrifugation at 12,900 *g* for 30 min at 4 °C, air-dried, than the pellet was redissolved in 750 μl of RNase-free water and transferred to a 2-ml tube. 1 ml of TRIzol (MRC, Inc.) was added, and subsequent purification of RNA was carried out according to the manufacturer’s instruction. All the isolated total RNAs were treated with DNase I to remove any DNA traces. Quantity and quality of isolated RNAs were estimated with a UV/VIS spectrophotometer (OD_260/280_ values for all the isolated RNAs were greater than 1.95). The assessment of RNA integrity was performed by denaturing RNA electrophoresis in TAE agarose gel according to Masek T. et al. [39].

About 2 μg of total RNA from each tissue was reverse transcribed to cDNA using 1 μM of oligodeoxythymidine (oligo dT_25_) primer. The synthesized cDNAs were diluted with sterile water up to 100-μl volume and used as the template for real-time RT-PCR. The intercalating EvaGreen Dye-based real-time RT-PCR was performed in a real-time Detection Thermal Cycler DTprime (DT-96) (DNA-Technology) under following conditions: 5 min at 95 °C followed by 50 cycles of 10 s at 95 °C, 30 s at 59 °C, and 40 s at 72 °C. The β-tubulin [GenBank accession number: X54844] gene was chosen as an internal control so that to normalize the amount of total RNA present in each reaction. It was shown that the β-tubulin gene had the most stable expression level among all the tested housekeeping genes in garden pea [40]. The primers were designed as follows: CCCGTGGAACTGTATCTGC (Ps-LTP1-Fw), GCCGTGGAACTGTATCCTG (Ps-LTP2-Fw); CCGGCGTGCAACTGTAATGA (Ps-LTP3-Fw); CGCGGCACTGATCTTGTAA (Ps-LTP1-3-Rv); GCTCCCAGCAGTACAGGACTCT (β-tubulin-Fw), TGGCATCCCACATTTGTTGA (β-tubulin-Rv). The reaction mixture (20 μl) contained 1 μl of EvaGreen Dye 20X solution (Biotium), 0.2 mM of each deoxynucleotide, 2 U of Taq DNA polymerase (Evrogen), 2 μl 10X buffer for Taq, 0.2 μM each of forward and reverse primers, and 1 μl of cDNA template (equivalent to 20 ng of total RNA).

### Tryptic digestion

Ps-LTP1 was reduced in the presence of 10 mM DTT and digested with modified trypsin (Promega) in 50 mM NH_4_HCO_3_, pH 8.0. The hydrolysis was performed at 37 °C for 1 h and stopped by addition of 0.5 % TFA in 10 % acetonitrile. Mass spectra of the protein fragments were measured in the positive-ion reflectron mode. MS/MS spectra of the Ps-LTP1 fragments were measured using Ultraflex MALDI-TOF/TOF mass spectrometer (Bruker).

### Reduction and alkylation of Ps-LTP1

The cysteine residues number was determined using iodoacetamide alkylation. The Ps-LTP1 sample (10 μg) was dissolved in 90 μl of 50 мМ NH_4_HCO_3_, pH 8.0, containing 2 mM dithiothreitol (DTT), and incubated at 37 °С for 2 h. Then the reduced protein was alkylated using 20 mM iodoacetamide for 1 h at room temperature in the dark. The S-carboxamidomethyl derivative was desalted immediately by RP-HPLC on Luna C_18_ column. The presence of disulfide bonds was demonstrated by the same scheme without prior reduction.

### Antimicrobial assay

Antimicrobial activity of Ps-LTP1 was measured by microspectrophotometry using 96-well microplates and serial dilutions of the protein as described [15]. The IC_50_ was defined as the lowest protein concentration that causes at least 50 % growth inhibition after 24 or 48 h of incubation in case of bacteria or fungi, respectively. Spore germination and morphology of hyphae were observed under Olympus CKX41 microscope after 12 and 24 h of spore suspension incubation (in half-strength potato glucose broth) at 25 °C in the presence of the protein solutions or water (as a control).

### Lipid binding

An ability of Ps-LTP1 to bind lipids was assessed using the fluorescent probe TNS as previously described [17]. Excitation and emission wavelengths were set at 320 and 437 nm, respectively. TNS (3.5 μM) with or without a lipid (18 μM; all FAs and JA from Sigma, lysolipids from Avanti) was incubated for 1 min in a stirred cuvette containing 1 ml of the measurement buffer (175 mM mannitol, 0.5 mM K_2_SO_4_, 0.5 mM CaCl_2_, 5 mM MES, pH 7.0) before the fluorescence was recorded (F_0_). Then Ps-LTP1 (4 μM) was added and 2 min later the fluorescence was recorded at equilibrium (F). The results were expressed as a percentage of Ps-LTP1–TNS complex fluorescence calculated according to [(F–F_0_)/F_C_] × 100 %, where F_C_ is the fluorescence of the Ps-LTP1–TNS complex in the absence of a lipid.

### Dye leakage from lipid vesicles

Calcein-entrapped LUVs composed of POPC or POPG or POPC/POPG (a 1:1 molar ratio) (all from Avanti) were prepared in the buffer containing 50 mM calcein, 10 mM HEPES, 200 mM NaCl, 0.5 mM EDTA, pH 7.5. The suspension was then extruded at room temperature through two stacked polycarbonate membrane filters of 100 nm (Nucleopore), 10 times through each pair, on a Mini-extruder (Avanti Polar Lipids, Alabaster, AL). Untrapped calcein was removed by gel filtration on a Sephadex G-75 column. The eluted calcein-entrapped vesicles were further diluted to the desired lipid concentration. The leakage of calcein from the LUVs was monitored by measuring fluorescence intensity at an excitation wavelength of 490 nm and an emission wavelength of 520 nm using F-2710 spectrofluorimeter (Hitachi). Maximum fluorescence intensity was determined by lysing vesicles in the presence of 1.5 % Triton X-100. The percentage of dye leakage caused by the protein was calculated as follows:

$$ \mathrm{Dye}\ \mathrm{leakage}\ \left(\ \%\right) = \left(\mathrm{F}\hbox{-} {\mathrm{F}}_0\right)/\left(\mathrm{F}\mathrm{t}\hbox{-} {\mathrm{F}}_0\right)\kern0.5em \mathrm{x}\kern0.5em 100\ \%, $$

where F is the fluorescence intensity after the protein addition, F_0_ and Ft are fluorescence intensities without the protein and with Triton X-100, respectively.

### Heterologous expression of unlabeled and ^13^C,^15^N-labeled Ps-LTP1 in *E. coli*

The recombinant plasmid pET-His8-TrxL-Ps-LTP1 (6033 bp) was constructed by ligating the 5253 bp BglII/XhoI fragment of pET-31b(+) vector (Novagen) with a PCR-constructed insert containing the T7 promoter, the ribosome binding site and the sequence encoding the recombinant protein that included octahistidine tag, TrxL carrier protein (*E. coli* thioredoxin A amplified from pET-32a(+) vector (Novagen) in which the Met37Leu mutation was introduced), methionine residue and the mature Ps-LTP1 sequence [GenBank accession number: KJ569141]. The last one was amplified from *P. sativum* cDNA pool using the mutagenic (Met11Leu) forward primer (GCGGGATCCATGGCTTTGTCTTGTGGAACTGTATCCGCTGATTTGGCTCCATGCGTTAC) and the reverse primer (GCGGAATTCTCAAAACCTAACAGTGTTACAG).

The culture of BL21 (DE3) Star™ cells transformed by pET-His8-TrxL-Ps-LTP1 was grown in LB media with addition of 100 μg/ml ampicillin and 20 mM glucose. After OD_600_ reached ~0.7, the cells were induced with 0.2 mM isopropyl-β-D-1-thiogalactopyranoside (IPTG) and incubated at 26 °C at 220 rpm for 4–6 h. ^13^C,^15^N-labeled Ps-LTP1 was expressed in M9 minimal medium containing 1 g/L ^15^NH_4_Cl (CIL) and 20 mM [U-^13^C_6_]-labeled D-glucose (CIL) as the sole nitrogen and carbon sources, respectively. After OD_600_ reached ~0.7, the cells were induced with 0.2 mM IPTG and further incubated at 26 °C at 220 rpm for 10–12 h. The expression level and solubility of the fusion protein were monitored using SDS-PAGE. The target protein was purified by sequential lysis of bacterial cells, immobilized metal ion affinity chromatography (IMAC), dialysis, cyanogen bromide cleavage of the fusion protein, repeated IMAC, and RP-HPLC.

### NMR experiments and spatial structure calculation

NMR investigation was done using 1.0 mM sample of the ^13^C,^15^N -labeled Ps-LTP1 in 5 % D_2_O at pH 5.5 and 30 °C. NMR spectra were acquired on Bruker Avance 700 spectrometer equipped with a room-temperature triple-resonance (^1^H, ^13^C, and ^15^N) probe. Backbone resonance assignment was obtained using the standard set of 3D triple-resonance experiments [41]. 3D ^13^C-HCCH-TOCSY and ^15^N- or ^13^C-filtered 3D TOCSY and NOESY spectra were used for side chains assignment. The ^3^J_H_^N^_H_^α^ and ^3^J_NH_^β^ coupling constants were measured using 3D HNHA and HNHB experiments [42]. ^3^J_C_^γ^_C’_, ^3^J_C_^γ^_N_ constants for Val, Ile, and Thr residues were quantitatively calculated from the cross-peak intensities in the spin-echo difference ^13^С-HSQC experiments [42]. Temperature coefficients of amide protons (Δδ^1^H^N^/ΔT) were measured over a temperature range 20–70 °C using 2D ^15^N-HSQC spectra. Heteronuclear ^15^N-{^1^H} NOE values were measured at 30 °C using pseudo 3D experiment [41].

Spatial structure calculation was performed in the CYANA 3.0 program [43]. Upper interproton distance constraints were derived from the intensities of cross-peaks in 3D ^15^N-NOESY-HSQC and ^13^С-NOESY-HSQC (τ_m_ = 80 ms) spectra via a “1/r^6^” calibration. ^1^H, ^13^C, and ^15^N backbone chemical shifts were used as an input for the TALOS+ software to predict the secondary structure [44]. Torsion angle restraints and stereospecific assignments were obtained from J coupling constants, NOE intensities and TALOS+ predictions. Hydrogen bonds were introduced basing on Δδ^1^H^N^/ΔT values. The disulfide bond connectivity pattern was established on the basis of the observed NOE contacts and verified during preliminary stages of the spatial structure calculation. The location and volume of the cavities in the proteins were calculated using CASTp with a 1.4 Å probe radius (http://sts.bioe.uic.edu/castp/calculation.php) [45]. The atomic coordinates for the major structural form of Ps-LTP1 have been deposited in the PDB under accession code 2N81.

### In silico analysis for the prediction of allergenicity

For prediction of the potential allergenicity, we used allergen online databases “The Food Allergy Research and Resource Program” (FARRP) (http://www.allergenonline.org), AllergenFP v.1.0 (http://ddg-pharmfac.net/AllergenFP/index.html), and Structural Database of Allergenic Proteins (SDAP) (https://fermi.utmb.edu/SDAP).

### Simulated gastrointestinal digestion of Ps-LTP1

Simulation of gastrointestinal digestion in vitro was performed as described in [46]. Briefly, gastric digestion was performed for 2 h using 50 ng of pepsin (Sigma) per 1 μg of the substrate in 0.1 M HCl, pH 2.0. For duodenal digestion, pH of the mixture resulting from gastric digestion was adjusted to 8.0 by addition of ammonium bicarbonate. The obtained mixture was incubated for 24 h at 37 °C with 2.5 ng of trypsin (Promega) and 10 ng of α-chymotrypsin (Sigma) per 1 μg of the substrate. Degree of proteolysis was monitored by SDS-PAGE. Gel images were analyzed with Gel-Pro software.

### Patients’ sera and immunoglobulin binding assays

Sera of 20 allergic individuals were collected at the Center for Molecular Diagnostics of the Central Research Institute of Epidemiology, Moscow, approved and in accordance with the rules and regulations of the institutional review board. Sera of 9 patients with food allergy were selected basing on the presence of specific IgE to Pru p 3 therein. Total IgE level was determined using Total IgE HRP EIA kit (Dr. Fooke) according to the manufacturer’s instruction*.* Specific IgE level was determined by ELISA assay. Plate wells (Costar, 3590) were coated with the recombinant proteins in TBS (20 mM Tris-HCl, 150 mM NaCl, pH 7.4) for 1 h at 37 °C, saturated with 1 % bovine serum albumin (BSA) in TBS buffer for 1 h at 37 °C, and then incubated with the serum pool from Pru p 3-sensitized patients (1:2 to 1:16 serial dilutions) for 2 h at 37 °C. Specific IgE binding was detected by using peroxidase-conjugated anti-human IgE from goat (Sigma) (1:2,000 dilution), and 3,3′,5,5′-tetramethylbenzidine (TMB) liquid substrate system for ELISA (Sigma). The enzymatic reaction was stopped after 20 min by acidification with 2 N H_2_SO_4_, and absorbance values were determined at 450 nm. TBS, containing 0.05 % Tween-20 (TBS-T), was used as washing solution on each step. Based on the level of IgE to Pru p 3 found in sera of nonalergic patients, the values <0.25 OD were considered as negative.

The interaction of previously obtained polyclonal rabbit anti-Len c 3 antiserum with recombinant proteins was evaluated by means of western blotting and ELISA assays. The serum obtained from the rabbit prior to immunization was used as negative control. The protein extract from pea seeds was obtained as described in the section ***“Methods. Isolation of pea LTP”***. ELISA and inhibition experiments with anti-Len c 3 rabbit antiserum were performed as described above with some modifications. After blocking of free binding sites, the plate wells were incubated with anti-Len c 3 rabbit antiserum in TBS (1:500 to 1:64,000 serial dilutions) for 1 h at 37 °C. In inhibition ELISA assays the anti-Len c 3 rabbit antiserum was preincubated with increasing quantities of the recombinant proteins for 3 h at room temperature and then added to the plates coated with the recombinant Len c 3. Peroxidase-conjugated anti-rabbit antibodies from goat (1:50,000 dilution) in TBS were used for detection.

### Availability of supporting data

The protein sequence data reported in this paper will appear in the UniProt Knowledgebase under the accession number **C0HJR7** (Ps-LTP1). The nucleotide sequences have been deposited in the GenBank under the accession numbers **KJ569141** (Ps-LTP1), **KJ569142** (Ps-LTP2), and **KJ569143** (Ps-LTP3)**.** The atomic coordinates and structure factors have been deposited in the Worldwide Protein Data Bank under the accession number PDB: **2N81**.

### Ethics approval

Sera from patients were collected at the Center for Molecular Diagnostics of the Central Research Institute of Epidemiology, Moscow, approved and in accordance with the rules and regulations of the institutional review board.

### Consent for publication

Not applicable.

## Additional files

Additional file 1: Purification by chromatographic methods. A. Cation-exchange chromatography on a HiTrap SP FF column and SDS-PAGE of Fraction 2. B. RP-HPLC on Luna C_18_ column and SDS-PAGE of Fraction 1 containing Ps-LTP1. M – molecular mass standards. (PNG 3156 kb) Additional file 2: MALDI-TOF-MS analysis. A. Ps-LTP prior the reaction with iodoacetamide. B. Ps-LTP1 modified with iodoacetamide after previous reduction (the calculated molecular mass is 9863.91 Da). (PNG 1776 kb) Additional file 3: CD-spectrum of Ps-LTP1. (PNG 648 kb) Additional file 4: Confirmation of primary structure of Ps-LTP1 by tryptic hydrolysis. A. Tryptic fragments of the mature Ps-LTP1. B. MALDI-LIFT-TOF/TOF mass spectrum of the cluster ion at *m/z* of 3560.447 recorded from a tryptic digest of the reduced.Ps-LTP1. (PNG 5470 kb) Additional file 5: Comparison of the measured and calculated molecular masses of tryptic fragments of the reduced Ps-LTP1. (DOCX 19 kb) Additional file 6: Percentage of calcein dye-leakage from POPG LUV upon addition of Ps-LTP1. (PNG 1485 kb) Additional file 7: A. ^15^N-HSQC spectrum of Ps-LTP1 (pH 5.5, 30 °C). The obtained resonance assignment for the major structural form of the protein is shown. Resonances of the minor form are marked with asterisks. Resonances of side chain groups are marked with superscripts denoting names of corresponding ^15^N atoms (δ, ε, or η). Resonances of Asn and Gln NH_2_ groups are connected by dotted lines. “Folded” resonances are underlined. Inset shows N^η^H resonances of guanidinium group of Arg47 for both the major and minor structural forms. B. The fragments of 80 ms 3D ^15^N-NOESY-HSQC spectrum showing exchange H^N^-H^N^ cross-peaks between the two structural forms of the protein. (PNG 3085 kb) Additional file 8: Statistics for the best CYANA structures of the major structural form of Ps-LTP1 in water at pH 5.5. (DOCX 18 kb) Additional file 9: Comparison of spatial structures of pea Ps-LTP1, lentil Lc-LTP2 [27], and peach Pru p 3 [28]. The structures were superimposed over C^α^ atoms of the eight conserved cysteine residues. Conservative Pro and Asp residues in helix H1 are shown. (PNG 2114 kb) Additional file 10: Thermal stability of the Ps-LTP1 spatial structure. A. Secondary chemical shifts of Ps-LTP1 ^1^H^N^ protons measured at two temperatures 20 °C and 70 °C. (Δδ = δ^obs^ – δ^r.c.^, where δ^obs^ – observed chemical shift and δ^r.c.^ – chemical shift in random coil conformation). The negative and positive values of Δδ^1^H^N^ correspond to α-helix and β-structural propensity, respectively [18]. Please not that at the high temperatures some backbone resonances became unobservable due to increase in the rate of the observed conformational exchange process (from slow to intermediate on the NMR time scale) or increase in the rate of amide protons exchange with water (from slow to intermediate or fast). The red arrows denote amide protons with temperature gradients less than −10.0 ppb/K, which exceed the temperature gradient of water protons. B. Dependence of Δδ^1^H^N^ values for several residues from the temperature. The non-linear behavior of Δδ^1^H^N^ for Val93 is due to change in the rate of the conformational exchange process. (PNG 3754 kb) Additional file 11: Allergenicity assessment *in silico* of Ps-LTP1. (DOCX 22 kb) Additional file 12: A. SDS-PAGE and B. immunoblotting with rabbit cross-reactive anti-Len c 3 antiserum. 1,2,3 – the recombinant Len c 3, Pru p 3, and Ps-LTP1, respectively; 4 – the pea extract. (BMP 1836 kb) Additional file 13: Immunoglobulin-binding assays with rabbit anti-Len c 3 antiserum. A. ELISA. B. Inhibition assays. (PNG 1966 kb)

## Abbreviations

DTT: dithiothreitol
EDTA: ethylenediaminetetraacetic acid
ELISA: enzyme-linked immunosorbent assay
FA: fatty acid
JA: jasmonic acid
LMPC: lyso-myristoyl phosphatidylcholine
LMPG: lyso-myristoyl phosphatidylglycerol
LPPC: lyso-palmitoyl phosphatidylcholine
LPPG: lyso-palmitoyl phosphatidylglycerol
LTP: lipid transfer protein
LUV: large unilamellar vesicle
MALDI-TOF-MS: time-of-flight matrix-assisted laser desorption ionization mass spectrometry
MES: 2-(N-Morpholino)ethanesulfonic acid
NMR: nuclear magnetic resonance
RACE: rapid amplification of cDNA ends
PMSF: phenylmethanesulfonyl fluoride
POPC: 1-Palmitoyl-2-oleoyl-sn-glycero-3-phosphocholine
POPG: 1-Palmitoyl-2-oleoyl-sn-glycero-3-phospho-(1'-rac-glycerol)
PVPP: polyvinylpolypyrrolidone
TFA: trifluoroacetic acid
TNS: 6-(p-Toluidino)-2-naphthalenesulfonic acid.
