# Supplementary figures and images for: A novel lipid transfer protein from the pea Pisum sativum: isolation, recombinant expression, solution structure, antifungal activity, lipid binding, and allergenic properties
Source: BMC Plant Biol. 2016 Apr 30;16:107. doi: 10.1186/s12870-016-0792-6 (PMC4852415; doi:10.1186/s12870-016-0792-6)

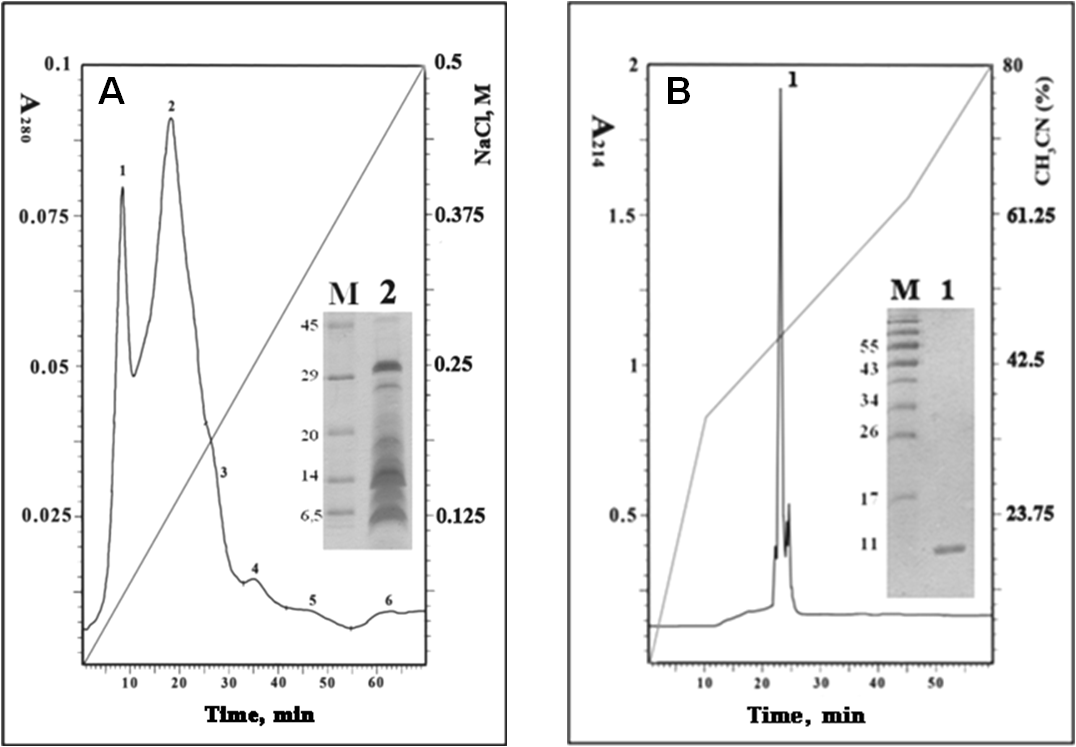

Supplement: Additional file 1: — Purification by chromatographic methods. A. Cation-exchange chromatography on a HiTrap SP FF column and SDS-PAGE of Fraction 2. B. RP-HPLC on Luna C18 column and SDS-PAGE of Fraction 1 containing Ps-LTP1. M – molecular mass standards. (PNG 3156 kb) [file 12870_2016_792_MOESM1_ESM.png]

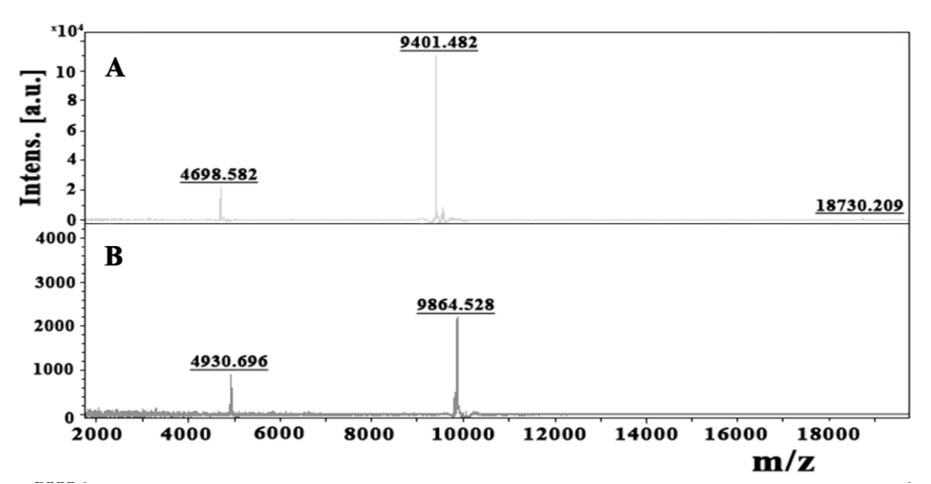

Supplement: Additional file 2: — MALDI-TOF-MS analysis. A. Ps-LTP prior the reaction with iodoacetamide. B. Ps-LTP1 modified with iodoacetamide after previous reduction (the calculated molecular mass is 9863.91 Da). (PNG 1776 kb) [file 12870_2016_792_MOESM2_ESM.png]

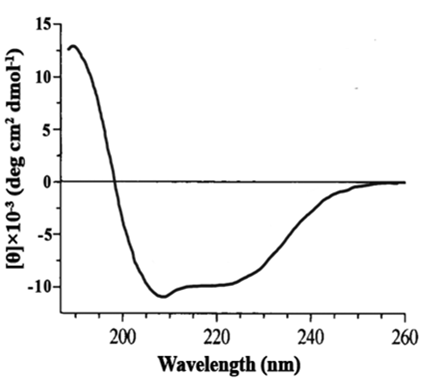

Supplement: Additional file 3: — CD-spectrum of Ps-LTP1. (PNG 648 kb) [file 12870_2016_792_MOESM3_ESM.png]

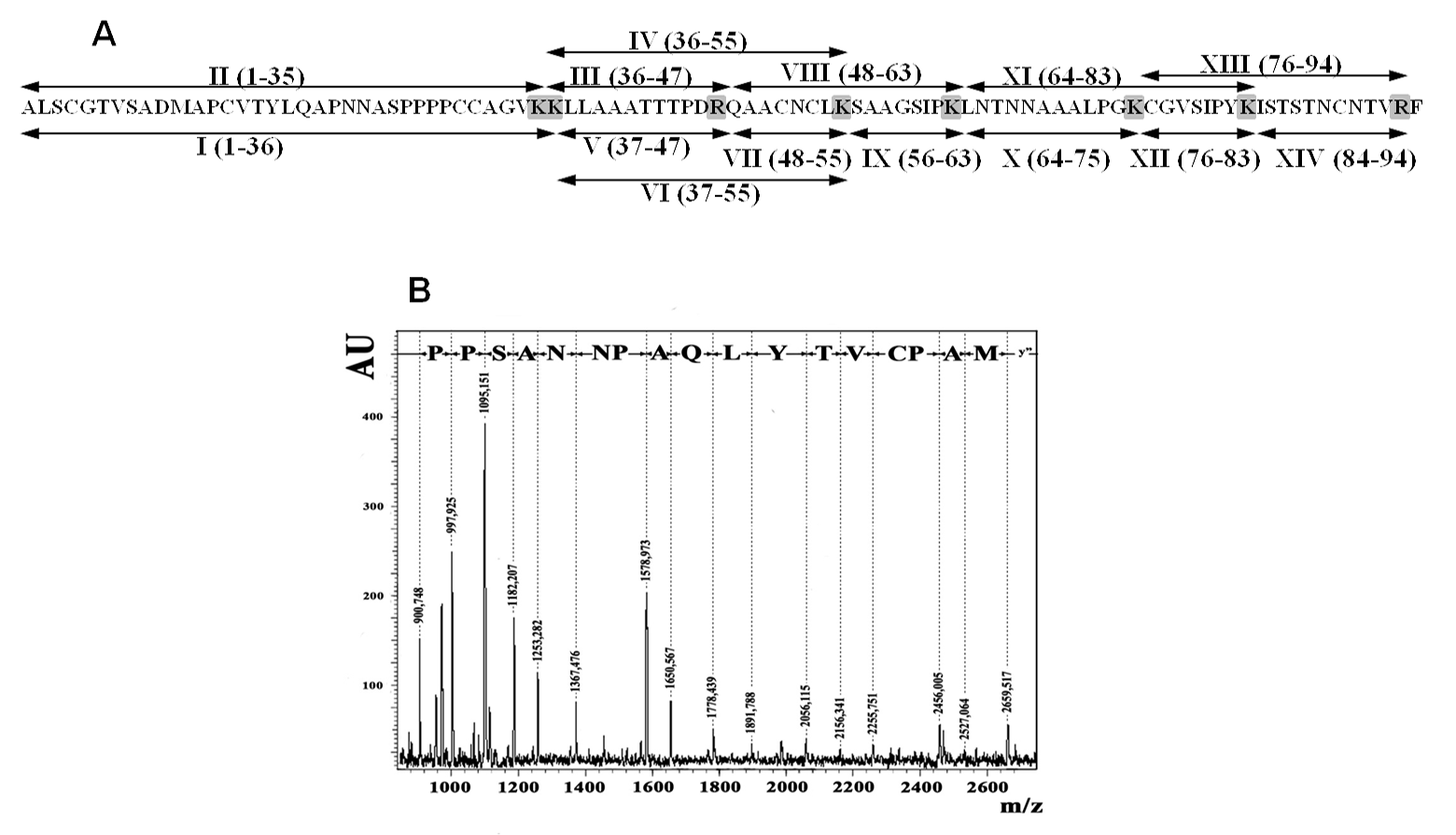

Supplement: Additional file 4: — Confirmation of primary structure of Ps-LTP1 by tryptic hydrolysis. A. Tryptic fragments of the mature Ps-LTP1. B. MALDI-LIFT-TOF/TOF mass spectrum of the cluster ion at m/z of 3560.447 recorded from a tryptic digest of the reduced.Ps-LTP1. (PNG 5470 kb) [file 12870_2016_792_MOESM4_ESM.png]

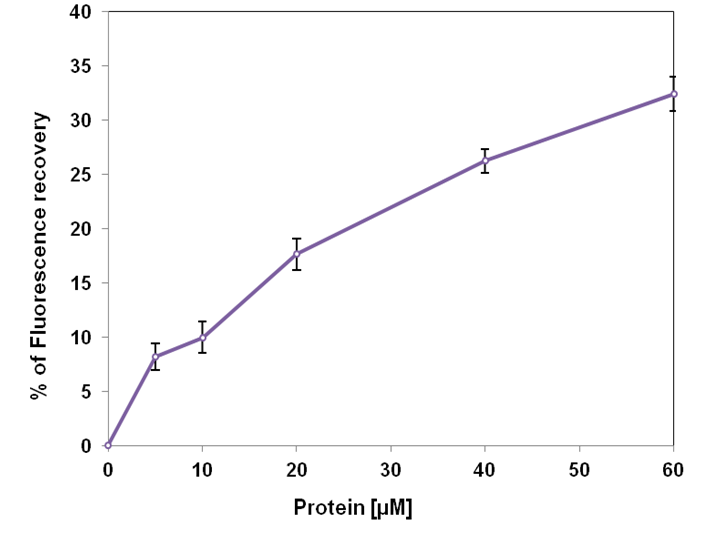

Supplement: Additional file 6: — Percentage of calcein dye-leakage from POPG LUV upon addition of Ps-LTP1. (PNG 1485 kb) [file 12870_2016_792_MOESM6_ESM.png]

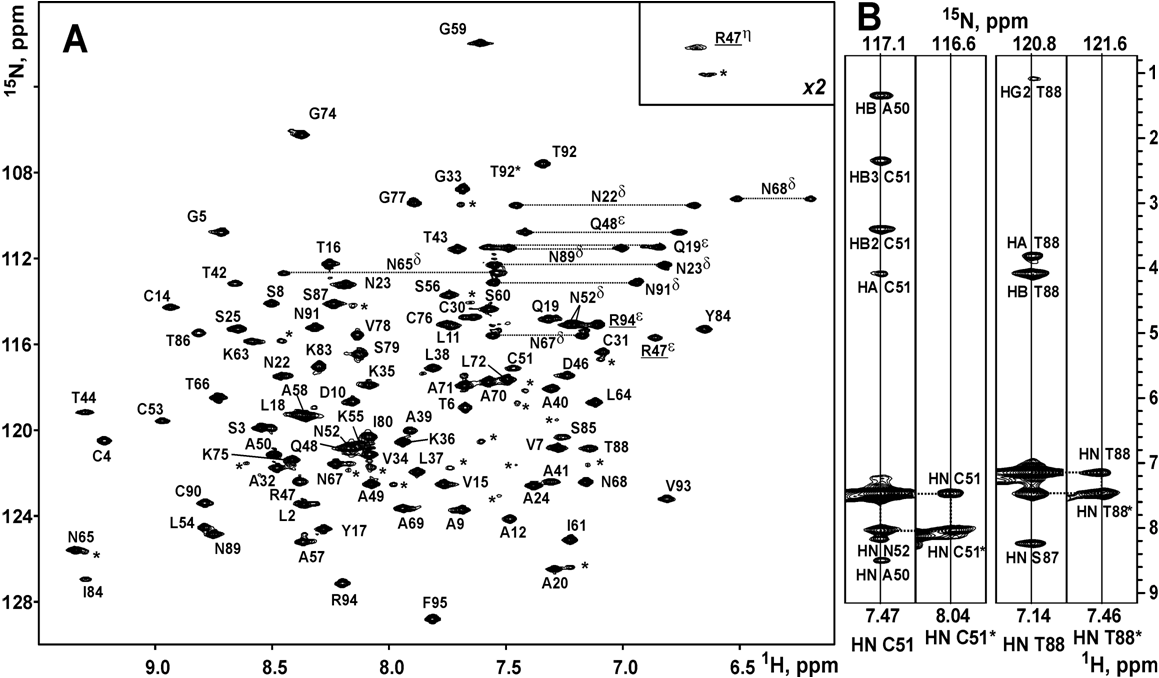

Supplement: Additional file 7: — A. 15N-HSQC spectrum of Ps-LTP1 (pH 5.5, 30 °C). The obtained resonance assignment for the major structural form of the protein is shown. Resonances of the minor form are marked with asterisks. Resonances of side chain groups are marked with superscripts denoting names of corresponding 15N atoms (δ, ε, or η). Resonances of Asn and Gln NH2 groups are connected by dotted lines. “Folded” resonances are underlined. Inset shows NηH resonances of guanidinium group of Arg47 for both the major and minor structural forms. B. The fragments of 80 ms 3D 15N-NOESY-HSQC spectrum showing exchange HN-HN cross-peaks between the two structural forms of the protein. (PNG 3085 kb) [file 12870_2016_792_MOESM7_ESM.png]

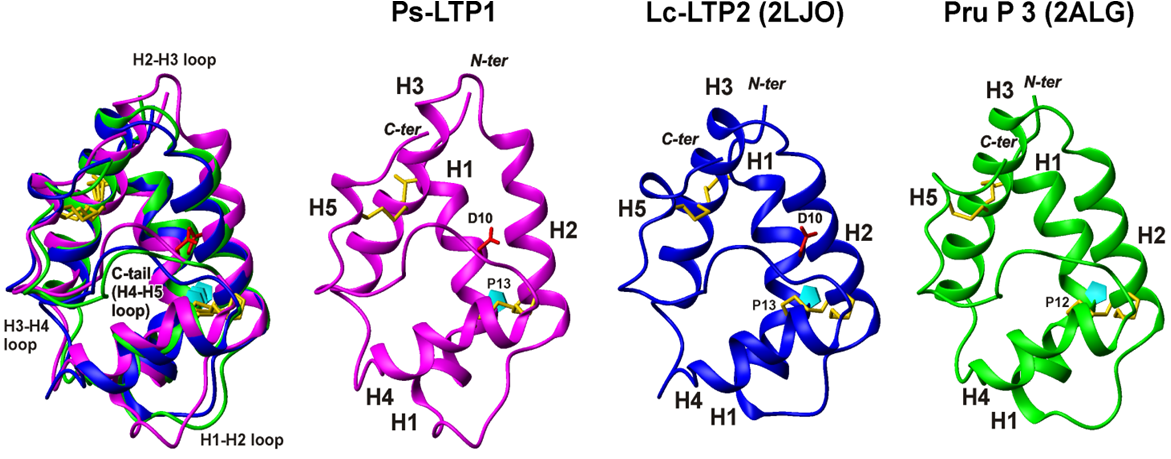

Supplement: Additional file 9: — Comparison of spatial structures of pea Ps-LTP1, lentil Lc-LTP2 [27], and peach Pru p 3 [28]. The structures were superimposed over Cα atoms of the eight conserved cysteine residues. Conservative Pro and Asp residues in helix H1 are shown. (PNG 2114 kb) [file 12870_2016_792_MOESM9_ESM.png]

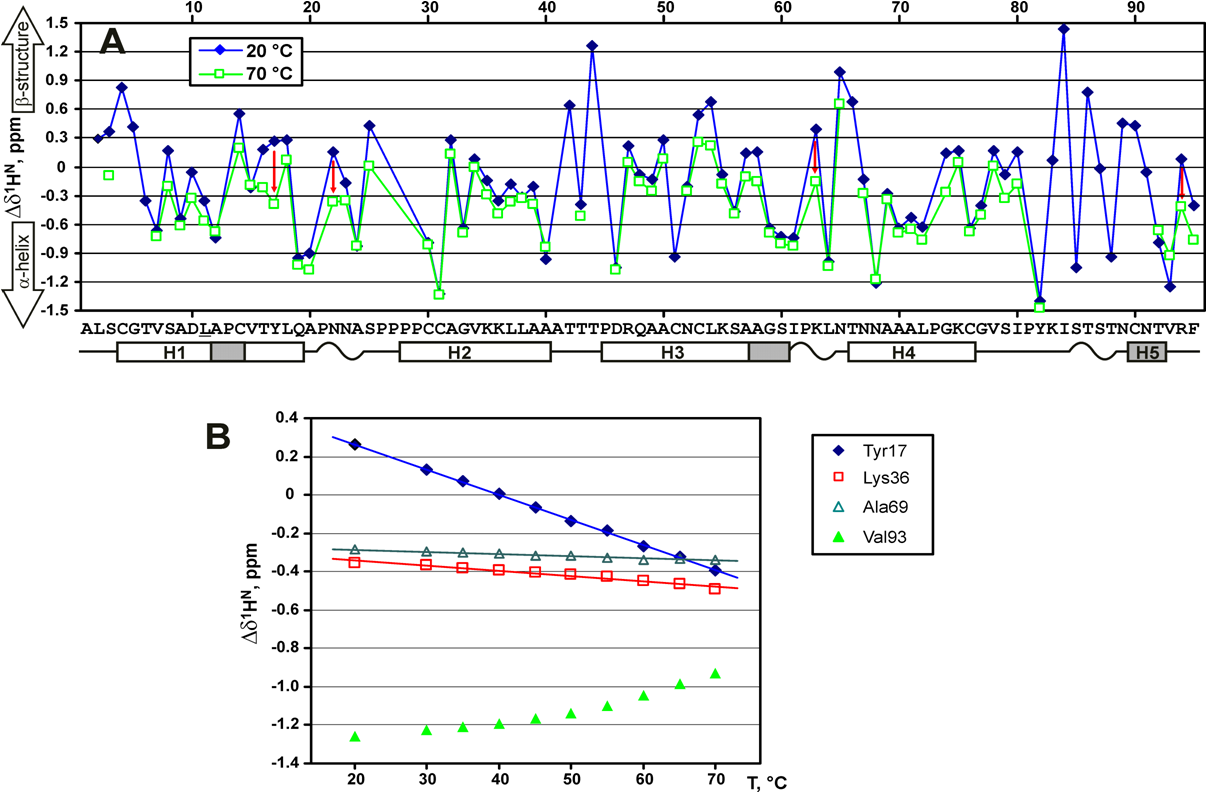

Supplement: Additional file 10: — Thermal stability of the Ps-LTP1 spatial structure. A. Secondary chemical shifts of Ps-LTP1 1HN protons measured at two temperatures 20 °C and 70 °C. (Δδ = δobs – δr.c., where δobs – observed chemical shift and δr.c. – chemical shift in random coil conformation). The negative and positive values of Δδ1HN correspond to α-helix and β-structural propensity, respectively [18]. Please not that at the high temperatures some backbone resonances became unobservable due to increase in the rate of the observed conformational exchange process (from slow to intermediate on the NMR time scale) or increase in the rate of amide protons exchange with water (from slow to intermediate or fast). The red arrows denote amide protons with temperature gradients less than −10.0 ppb/K, which exceed the temperature gradient of water protons. B. Dependence of Δδ1HN values for several residues from the temperature. The non-linear behavior of Δδ1HN for Val93 is due to change in the rate of the conformational exchange process. (PNG 3754 kb) [file 12870_2016_792_MOESM10_ESM.png]

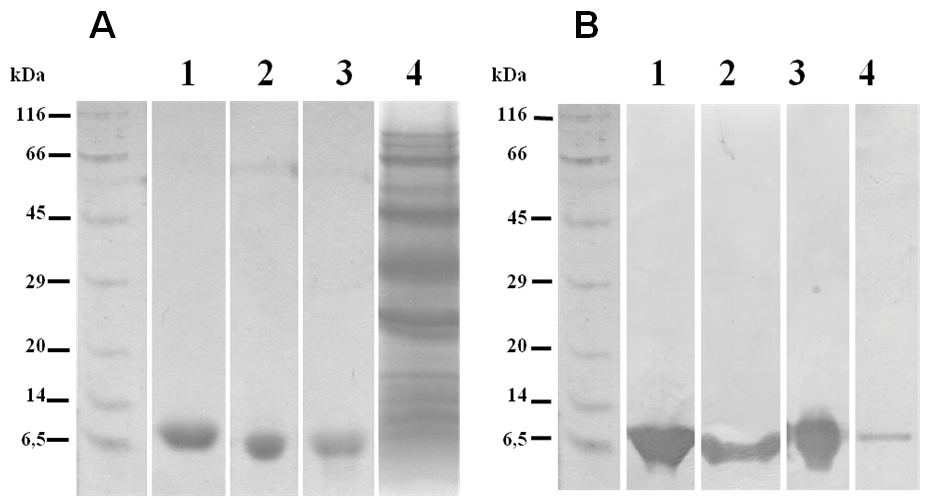

Supplement: Additional file 12: — A. SDS-PAGE and B. immunoblotting with rabbit cross-reactive anti-Len c 3 antiserum. 1,2,3 – the recombinant Len c 3, Pru p 3, and Ps-LTP1, respectively; 4 – the pea extract. (BMP 1836 kb) [file 12870_2016_792_MOESM12_ESM.bmp]

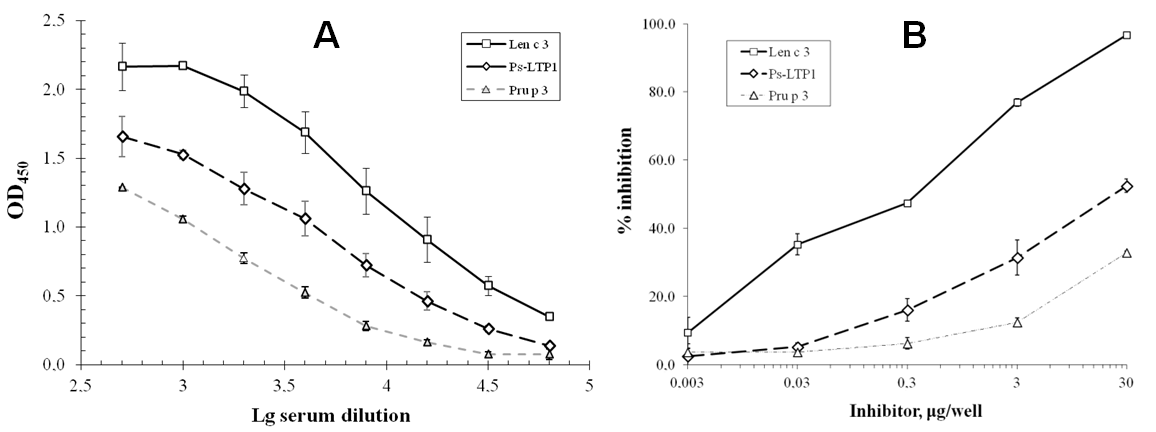

Supplement: Additional file 13: — Immunoglobulin-binding assays with rabbit anti-Len c 3 antiserum. A. ELISA. B. Inhibition assays. (PNG 1966 kb) [file 12870_2016_792_MOESM13_ESM.png]
