# Supplementary material for: A novel lipid transfer protein from the pea Pisum sativum: isolation, recombinant expression, solution structure, antifungal activity, lipid binding, and allergenic properties
Source: BMC Plant Biol. 2016 Apr 30;16:107. doi: 10.1186/s12870-016-0792-6 (PMC4852415; doi:10.1186/s12870-016-0792-6)
Supplement: Additional file 5: — Comparison of the measured and calculated molecular masses of tryptic fragments of the reduced Ps-LTP1. (DOCX 19 kb) [file 12870_2016_792_MOESM5_ESM.docx]

**Comparison of measured and calculated masses of reduced pea LTP tryptic fragments.**

| Fragment | Mass, daltons | |
| --- | --- | --- |
|  | measured | calculated |
| **I (1-35)** | 3560.447 | 3561.666 |
| **II (1-36)** | 3431.350 | 3433.571 |
| **III (36-47)** | 1257.743 | 1257.716 |
| **IV (36-55)** | 2088.025 | 2089.089 |
| **V (37-47)** | 1129.652 | 1129.621 |
| **VI (37-55)** | 1960.979 | 1960.994 |
| **VII (48-55)** | 850.380 | 850.391 |
| **VIII (48-63)** | 1560.701 | 1561.782 |
| **IX (56-63)** | 730.385 | 730.409 |
| **X (64-75)** | 1183.649 | 1183.642 |
| **XI (64-83)** | 2032.017 | 2031.069 |
| **XII (76-83)** | 866.438 | 866.444 |
| **XIII (76-94)** | 2042.033 | 2042.999 |
| **XIV (84-94)** | 1195.609 | 1195.573 |
