# Supplementary material for: A novel lipid transfer protein from the pea Pisum sativum: isolation, recombinant expression, solution structure, antifungal activity, lipid binding, and allergenic properties
Source: BMC Plant Biol. 2016 Apr 30;16:107. doi: 10.1186/s12870-016-0792-6 (PMC4852415; doi:10.1186/s12870-016-0792-6)
Supplement: Additional file 8: — Statistics for the best CYANA structures of the major structural form of Ps-LTP1 in water at pH 5.5. (DOCX 18 kb) [file 12870_2016_792_MOESM8_ESM.docx]

**Statistics for the best CYANA structures of the major structural form of Ps-LTP1 in water at pH 5.5.**

| **Distance and angle restraints** |  |
| --- | --- |
| Total NOE contacts | 623 |
| intraresidual | 242 |
| sequential (\|i-j\|=1) | 223 |
| medium-range (1<\|i-j\|<4) | 106 |
| long-range (\|i-j\|>4) | 52 |
| Hydrogen bonds restraints (50 bonds, upper/lower) | 100/100 |
| S-S bond restraints (4 bonds, upper/lower) | 16/16 |
| Torsion angle restraints | 114 |
| Angle φ | 70 |
| Angle χ1 | 44 |
| **Total restraints/per residue:** | 969/10.2 |
| **Statistics for calculated structures** |  |
| Structures calculated/selected | 300/20 |
| CYANA target function (Å^2^) | 2.84± 0.02 |
| Violations of restraints |  |
| Distance (>0.2 Å) | 0 |
| Dihedral angles (>5 °) | 0 |
| RMSD (Å) overall (Ala1-Phe95) | |
| Backbone | 1.03±0.20 |
| Heavy atoms | 1.59±0.27 |
| RMSD (Å) H1-H4 helices (Cys4-Cys76) | |
| Backbone | 0.78±0.16 |
| Heavy atoms | 1.23±0.15 |
| Ramachandran analysis | |
| Residues in favored regions (%) | 88.9 |
| Residues in allowed regions (%) | 100 |
