# Supplementary material for: A novel lipid transfer protein from the pea Pisum sativum: isolation, recombinant expression, solution structure, antifungal activity, lipid binding, and allergenic properties
Source: BMC Plant Biol. 2016 Apr 30;16:107. doi: 10.1186/s12870-016-0792-6 (PMC4852415; doi:10.1186/s12870-016-0792-6)
Supplement: Additional file 11: — Allergenicity assessment in silico of Ps-LTP1. (DOCX 22 kb) [file 12870_2016_792_MOESM11_ESM.docx]

**Allergenicity assessment *in silico* of Ps-LTP1.**

| No. | Allergen ID, acc. No. | 80-mer  window^a^ | No. of exact 6-mer wordmatches^b^ | Full length^c^ | E-value |
| --- | --- | --- | --- | --- | --- |
| 1 | Len c 3, A0AT29 | 32 | 81.25 | 76.84 | 2.9e-27 |
| 2 | Pha v 3 b, ADC80503 | 20 | 67.50 | 63.54 | 2.8e-22 |
| 3 | Ara h 9 b, B6CG41 | 17 | 68.75 | 65.96 | 9.3e-23 |
| 4 | Ara h 9 a, B6CEX8 | 17 | 67.50 | 63.83 | 6.8e-23 |
| 5 | Pru p 3, P81402 | 16 | 60.00 | 58.06 | 3.2e-21 |
| 6 | Pyr c 3, Q9M5X6 | 15 | 58.75 | 56.99 | 8.4e-21 |
| 7 | Mor n 3, P85894 | 10 | 63.75 | 60.22 | 1.5e-19 |
| 8 | Rub i 3, Q0Z8V0 | 9 | 62.50 | 59.57 | 2.7e-20 |
| 9 | Pha v 3 a, ADC80502 | 9 | 60.00 | 55.44 | 1.4e-19 |
| 10 | Fra a 3 a, Q8VX12 | 9 | 60.00 | 55.91 | 9.5e-20 |
| 11 | Pru d 3, P82534 | 9 | 58.75 | 56.99 | 1.2e-20 |
| 12 | Pru ar 3, P81651 | 9 | 58.75 | 56.99 | 4.1e-20 |
| 13 | Cit s 3 b, Q6EV47 | 8 | 66.67 | 62.77 | 4.6e-21 |
| 14 | Fra a 3 c, Q4PLU0 | 8 | 58.75 | 54.84 | 3.4e-19 |
| 15 | Mal d 3, Q9M5X7 | 8 | 58.75 | 55.91 | 3.0e-20 |
| 16 | Pru av 3, Q9M5X8 | 8 | 56.25 | 55.91 | 1.3e-20 |
| 17 | Fra a 3 d, Q4PLT6 | 7 | 57.50 | 54.26 | 5.2e-19 |
| 18 | Hev b 12, Q8RYA8 | 6 | 58.75 | 54.84 | 2.9e-19 |
| 19 | Zea m 14 b, P19656-1 | 5 | 56.25 | 54.95 | 4.0e-18 |
| 20 | Zea m 14 a, P19656-2 | 5 | 56.25 | 54.84 | 2.2e-18 |
| 21 | Fra a 3 b, Q4PLT9 | 4 | 61.25 | 56.99 | 5.1e-20 |
| 22 | Cor a 8, Q9ATH2 | 4 | 61.25 | 55.32 | 2.1e-18 |
| 23 | Sin a 3, ABU95411 | 4 | 60.00 | 60.64 | 3.5e-19 |
| 24 | Pla or 3, A9YUH6 | 4 | 55.00 | 52.13 | 2.8e-17 |
| 25 | Pru du 3, C0L0I5 | 3 | 55.00 | 52.08 | 1.4e-17 |
| 26 | Art v 3 c, C4MGH0 | 3 | 55.00 | 52.69 | 7.5e-17 |
| 27 | Art v 3 b, C4MGG9 | 3 | 53.75 | 50.54 | 6.2e-16 |
| 28 | Lyc e 3, P93224 | 2 | 57.50 | 56.38 | 7.6e-18 |
| 29 | Api g 2, ACV04796 | 2 | 51.25 | 48.91 | 1.1e-15 |
| 30 | Art v 3 d, C4MGH1 | 2 | 48.75 | 47.87 | 1.1e-15 |
| 31 | Hel a 3, P82007 | 1 | 47.50 | 43.48 | 1.2e-14 |

^a^ Percent of identical amino acids in the aligned 80-aa sliding window.

^b^ The number of exact 6 a.a. hits the input sequence had with this allergen.

^c^ Results of FASTA alignment of the complete input sequence against UniProt and WHO-IUIS database, percentage identity.
